# Safety and immunogenicity of a candidate Middle East respiratory syndrome coronavirus viral-vectored vaccine: a dose-escalation, open-label, non-randomised, uncontrolled, phase 1 trial

**DOI:** 10.1016/S1473-3099(20)30160-2

**Published:** 2020-07

**Authors:** Pedro M Folegatti, Mustapha Bittaye, Amy Flaxman, Fernando Ramos Lopez, Duncan Bellamy, Alexandra Kupke, Catherine Mair, Rebecca Makinson, Jonathan Sheridan, Cornelius Rohde, Sandro Halwe, Yuji Jeong, Young-Shin Park, Jae-Ouk Kim, Manki Song, Amy Boyd, Nguyen Tran, Daniel Silman, Ian Poulton, Mehreen Datoo, Julia Marshall, Yrene Themistocleous, Alison Lawrie, Rachel Roberts, Eleanor Berrie, Stephan Becker, Teresa Lambe, Adrian Hill, Katie Ewer, Sarah Gilbert

**Affiliations:** a The Jenner Institute, Nuffield Department of Medicine, University of Oxford, Oxford, UK; b Institute of Virology, Philipps University of Marburg, Marburg, Germany; c German Center for Infection Research, Thematic Translational Unit Emerging Infections, Marburg, Germany; d International Vaccine Institute, Science Unit, Seoul, South Korea

## Abstract

**Background:**

Cases of Middle East respiratory syndrome coronavirus (MERS-CoV) infection continue to rise in the Arabian Peninsula 7 years after it was first described in Saudi Arabia. MERS-CoV poses a significant risk to public health security because of an absence of currently available effective countermeasures. We aimed to assess the safety and immunogenicity of the candidate simian adenovirus-vectored vaccine expressing the full-length spike surface glycoprotein, ChAdOx1 MERS, in humans.

**Methods:**

This dose-escalation, open-label, non-randomised, uncontrolled, phase 1 trial was done at the Centre for Clinical Vaccinology and Tropical Medicine (Oxford, UK) and included healthy people aged 18–50 years with negative pre-vaccination tests for HIV antibodies, hepatitis B surface antigen, and hepatitis C antibodies (and a negative urinary pregnancy test for women). Participants received a single intramuscular injection of ChAdOx1 MERS at three different doses: the low-dose group received 5 × 10^9^ viral particles, the intermediate-dose group received 2·5 × 10^10^ viral particles, and the high-dose group received 5 × 10^10^ viral particles. The primary objective was to assess safety and tolerability of ChAdOx1 MERS, measured by the occurrence of solicited, unsolicited, and serious adverse events after vaccination. The secondary objective was to assess the cellular and humoral immunogenicity of ChAdOx1 MERS, measured by interferon-γ-linked enzyme-linked immunospot, ELISA, and virus neutralising assays after vaccination. Participants were followed up for up to 12 months. This study is registered with ClinicalTrials.gov, NCT03399578.

**Findings:**

Between March 14 and Aug 15, 2018, 24 participants were enrolled: six were assigned to the low-dose group, nine to the intermediate-dose group, and nine to the high-dose group. All participants were available for follow-up at 6 months, but five (one in the low-dose group, one in the intermediate-dose group, and three in the high-dose group) were lost to follow-up at 12 months. A single dose of ChAdOx1 MERS was safe at doses up to 5 × 10^10^ viral particles with no vaccine-related serious adverse events reported by 12 months. One serious adverse event reported was deemed to be not related to ChAdOx1 MERS. 92 (74% [95% CI 66–81]) of 124 solicited adverse events were mild, 31 (25% [18–33]) were moderate, and all were self-limiting. Unsolicited adverse events in the 28 days following vaccination considered to be possibly, probably, or definitely related to ChAdOx1 MERS were predominantly mild in nature and resolved within the follow-up period of 12 months. The proportion of moderate and severe adverse events was significantly higher in the high-dose group than in the intermediate-dose group (relative risk 5·83 [95% CI 2·11–17·42], p<0·0001) Laboratory adverse events considered to be at least possibly related to the study intervention were self-limiting and predominantly mild in severity. A significant increase from baseline in T-cell (p<0·003) and IgG (p<0·0001) responses to the MERS-CoV spike antigen was observed at all doses. Neutralising antibodies against live MERS-CoV were observed in four (44% [95% CI 19–73]) of nine participants in the high-dose group 28 days after vaccination, and 19 (79% [58–93]) of 24 participants had antibodies capable of neutralisation in a pseudotyped virus neutralisation assay.

**Interpretation:**

ChAdOx1 MERS was safe and well tolerated at all tested doses. A single dose was able to elicit both humoral and cellular responses against MERS-CoV. The results of this first-in-human clinical trial support clinical development progression into field phase 1b and 2 trials.

**Funding:**

UK Department of Health and Social Care, using UK Aid funding, managed by the UK National Institute for Health Research.

**Research in context**

**Evidence before this study**

There are currently no licensed vaccines to prevent Middle East respiratory syndrome (MERS) or specific therapeutics to treat it. ChAdOx1 MERS has been previously reported to be immunogenic and protective in mice in a challenge model, and immunogenic and partially protective in dromedary camels in a natural transmission model. We searched PubMed for research articles published between database inception and Nov 20, 2019, using various combinations of the terms “MERS”, MERS-CoV”, “Middle East respiratory syndrome”, “anti-Middle East respiratory syndrome”, “vaccine”, “phase” and “clinical trial”. No language restriction was applied. One clinical trial has been published, describing a phase 1 study done in the USA, of a DNA vaccine against MERS, using a three-dose vaccination regimen of intramuscular injection followed by colocalised intramuscular electroporation at weeks 0, 4, and 12. The vaccine was well tolerated. Seroconversion measured by S1 ELISA occurred in 59 (86%) of 69 participants after two vaccinations and in 61 (94%) of 65 participants after three vaccinations. Neutralising antibodies were detected in 34 (50%) of 68 participants.

**Added value of this study**

This study is the first clinical study of ChAdOx1 MERS. At all dose levels tested (5 × 10^9^, 2·5 × 10^10^, and 5 × 10^10^ viral particles) the vaccine was safe and well tolerated. In the majority of participants, humoral and cellular MERS coronavirus (MERS-CoV)-specific immune responses were induced, and maintained at levels higher than the pre-vaccination response during the 1-year follow-up period. The study was done in the UK.

**Implications of all the available evidence**

A vaccine against MERS-CoV could be used to prevent zoonotic transmission, especially in people who are frequently exposed to camels in the Middle East, to immunise health-care workers in regions where hospital outbreaks have occurred, or to respond to an outbreak in a health-care setting or community. The immune correlates of protection against MERS-CoV have not yet been determined in any species. Immunisation with ChAdOx1 MERS results in rapid induction of immune responses against MERS-CoV, which are maintained for at least 1 year, and might therefore have value in preventing or limiting outbreaks in endemic regions. Further clinical studies, especially in endemic regions, should be done with this vaccine.

## Introduction

The Middle East respiratory syndrome coronavirus (MERS-CoV) causes an emerging zoonotic viral respiratory disease that was first described in 2012 and is now endemic in Saudi Arabia.^1^ Clinical presentation of MERS-CoV infections varies from asymptomatic to severe acute respiratory distress and death. MERS-CoV has since spread to different countries in the Middle East and other regions, with 2519 laboratory-confirmed cases of MERS-CoV infection, including 866 deaths in 27 countries, reported by Jan 31, 2020.^2^ MERS-CoV poses a major threat to public health security because of its epidemic potential and absence of currently available effective countermeasures, and it has been listed as a priority pathogen for research and development by WHO and other health agencies around the globe. Dromedary camels are now a recognised source of zoonotic infections, and occupational exposure has been associated with seroconversion,^3^ although only 40% of primary cases have been associated with direct camel exposure.^4^ Human-to-human transmission, especially in hospital environments, has been responsible for the majority of cases seen in outbreaks in the past 7 years.^5^ However, no sustained human-to-human transmission has been recorded so far (overall R_0_<1), and the role of asymptomatic or mild cases in transmission patterns remains controversial and unclear. No specific treatment options or licensed vaccines are currently available.

The non-specific clinical features of MERS often lead to delayed diagnosis and increased exposure in health-care facilities, contributing to the persistence of nosocomial outbreaks, which is further complicated by the absence of effective treatment options and suboptimal adherence to infection prevention and control and isolation practices.^6^ Considering the complexities around the implementation of overall control measures for MERS-CoV and the numerous challenges in filling knowledge gaps required to achieve it, vaccination remains the key cost-effective strategy to tackle the global MERS-CoV threat.

Coronaviruses are spherical, enveloped, large, positive-sense, single-stranded RNA genomes. A fourth of their genome is responsible for coding structural proteins, such as the spike glycoprotein, and envelope, membrane, and nucleocapsid proteins. Envelope, membrane, and nucleocapsid proteins are mainly responsible for virion assembly, whereas the spike glycoprotein is involved in receptor binding, mediating virus entry into host cells during infection via different receptors.^7^ MERS-CoV belongs to the phylogenetic lineage C of the genus Betacoronavirus, and it recognises the dipeptidyl peptidase 4 (DPP4) host receptor, which is well conserved between camels and humans.^8^ It is the sixth coronavirus known to cause human infections and the first human virus within lineage C. Severe acute respiratory syndrome coronavirus (SARS-CoV), which was responsible for the 2002–03 SARS global epidemic and SARS coronavirus 2 (SARS-CoV-2), which is responsible for the current pandemic, belong to lineage B (genus Betacoronavirus). HCoV-OC43 and HCoV-HKU1 are representatives of lineage A (genus Betacoronavirus), whereas HCoV-229E and HCoV-NL63 belong to the genus Alphacoronavirus. HCoV-OC43, HCoV-HKU1, HCoV-229E, and HCoV-NL63 are globally distributed and generally associated with mild respiratory symptoms, accounting for up to a third of all common cold cases.^9^

Spike is a type I, trimeric, transmembrane glycoprotein located at the surface of the viral envelope of coronaviruses, which can be divided into two functional subunits: the N-terminal S1 and the C-terminal S2. S1 and S2 on MERS-CoV are responsible for binding to the host cellular receptor DPP4 via the virus receptor-binding domain (RBD) and fusion of virus and host cell membranes, thereby mediating the entry of MERS-CoV into target cells.^7^ The RBD of MERS-CoV has a core structure, which is homologous to that of SARS-CoV, and a receptor-binding motif, which is specific to MERS-CoV, determining receptor recognition and viral pathogenesis.^10^ The roles of the spike protein in receptor binding and membrane fusion make it an ideal target for vaccine and antiviral development because it is the main target for neutralising antibodies.^11^

ChAdOx1 MERS consists of the replication-deficient simian adenovirus vector ChAdOx1, which has been described elsewhere,^12^ expressing a codon-optimised coding sequence for the full-length spike protein (S1 and S2 subunits) of the MERS-CoV isolate Camel/Qatar_2_2014 (GenBank, accession number KJ650098.1), including a 32 amino acid N-terminal tissue plasminogen activator leader sequence. Preclinical work has shown that the vaccine is highly immunogenic in animal models, being able to induce both cellular and humoral responses (polyfunctional CD8 T cells and neutralising antibodies).^13^ A single dose of ChAdOx1 MERS also conferred protective efficacy in transgenic mice expressing human DPP4 against a lethal MERS-CoV challenge, which supported progression into clinical development.^14^ Immunogenicity and partial protective efficacy in a natural transmission model in dromedary camels have also been reported.^15^ We aimed to assess the safety and immunogenicity of the ChAdOx1 MERS candidate MERS-CoV vaccine in humans.

## Methods

### Study design and participants

This dose-escalation, open-label, non-randomised, uncontrolled, phase 1 trial was done at the Centre for Clinical Vaccinology and Tropical Medicine (Oxford, UK). Participants were recruited through advertisements. Healthy people aged 18–50 years with negative pre-vaccination tests for HIV antibodies, hepatitis B surface antigen, and hepatitis C antibodies were eligible to participate. A negative urinary pregnancy test was required at screening and immediately before enrolment for all women. Full details of the eligibility criteria are described in the trial protocol provided in the appendix (p 42).

Written informed consent was obtained from all participants, and the trial was done in accordance with the principles of the Declaration of Helsinki and Good Clinical Practice. This study was approved in the UK by the Medicines and Healthcare Products Regulatory Agency (reference 21584/0381/001-0001) and the South Central—Oxford A Research Ethics Committee (reference 17/SC/0552). Vaccine use was authorised by the Genetically Modified Organisms Safety Committee of the Oxford University Hospitals National Health Service Trust (reference number GM462.18.101). An independent local safety monitor provided safety oversight.

### Procedures

The recombinant adenovirus was produced as previously described.^13^ The vaccine was manufactured according to current Good Manufacturing Practice by the Clinical Biomanufacturing Facility (University of Oxford, Oxford, UK) in a Tet-repressed HEK 293 cell line. The vectored vaccine was purified and sterile filtered to generate a clinical lot at a concentration of 1·74    × 10^11^ viral particles per mL.

ChAdOx1 MERS was administered as a single intramuscular injection into the deltoid at 5 × 10^9^ viral particles (low-dose group), 2·5 × 10^10^ viral particles (intermediate-dose group), and 5 × 10^10^ viral particles (high-dose group). A staggered-enrolment approach was used for the first three participants in each group, and interim safety reviews were done before dose escalation (details provided in the appendix p 49).

Blood samples were drawn and clinical assessments were done for safety as well as immunology endpoints before vaccination at day 0 and subsequently at days 2, 7, 14, 28, 56, 182, and 364 after vaccination. Participants were observed in the clinic for 1 h after the vaccination procedure and were asked to record any adverse events using electronic diaries during the 28-day follow-up period. Swelling at the injection site was objectively assessed by a member of the study team during the study visits.

Solicited (ie, expected and defined in the protocol) local site reactions (injection site pain, warmth, redness, swelling, and pruritus) and systemic symptoms (malaise, myalgia, arthralgia, fatigue, nausea, headache, feverishness, and fever, with a temperature of 37·5°C or higher) were recorded for 7 days by members of the study team and participants. Unsolicited adverse events were all other events not defined as solicited and were recorded for 28 days, and serious adverse events were recorded throughout the follow-up period.

Severity of adverse events was graded with the following criteria: mild (mild symptoms with no limitation to usual activity), moderate (mild-to-moderate limitation in usual activity), and severe (considerable limitation in activity, medication or medical attention required). Unsolicited adverse events were reviewed for causality by an independent clinician, and events considered to be possibly, probably, or definitely related to the study vaccine were reported. Laboratory adverse events were graded by use of site-specific toxicity tables, which were adapted from the US Food and Drug Administration toxicity grading scale.

Total anti-MERS spike IgG, to determine humoral immunity, was measured using a standardised in-house indirect ELISA and MERS-CoV neutralising antibodies. Nunc MaxiSorp 96-well plates (Life Technologies, Paisley, UK) were coated with 1 μg/mL of full-length recombinant clamp MERS spike protein (supplied by Keith Chappell, The School of Chemistry and Molecular Biosciences, The University of Queensland, Brisbane, QLD, Australia) in phosphate buffered saline and incubated at 4°C for 18 h overnight. The coated plates were washed six times with phosphate buffered saline-Tween and then blocked with casein for 1 h at room temperature. Plasma samples diluted to fall within the linear range of the curve (typically 1/500 in casein) were then added to individual wells on the plates. This step was followed by incubation of the plates at room temperature for 2 h and washing of the plates, as initially described. The plates were then incubated at room temperature for 1 h with a secondary antibody, alkaline phosphatase-conjugated goat anti-human IgG (γ-chain specific). After a final wash, plates were developed by adding 4-nitrophenyl phosphate in diethanolamine substrate buffer (Fisher Scientific UK, Loughborough, UK). The standard curve used on each plate was derived from a pool of volunteers' sera containing high-titre anti-MERS IgG. Endpoint titre determined by ELISA was used to identify the volunteer samples with the highest anti-MERS IgG titres after vaccination. A 1/100 dilution of the standard pool was used in a two-fold serial dilution to produce ten standard points that were assigned arbitrary ELISA units. The optical density values of the standard points were fitted to a four-parameter hyperbolic curve against the arbitrary ELISA units using GEN5 software (version 3.04; BioTek Instruments, Winooski, VT, USA), and the parameters estimated from the standard curve were used to convert absorbance values of individual test samples into ELISA units. Each ELISA plate consists of the samples and internal positive control (1/800 dilution of the standard pool, corresponding to standard 4) in triplicates, ten standard points in duplicates, and four blank wells. The optical density reading of the plates at 405 nm was done with an ELx808 microplate reader (BioTek Instruments).

Neutralising antibodies against live MERS-CoV were measured by use of paired serum samples obtained at day 0 and day 28. Induction of virus-neutralising antibodies was confirmed according to previously published protocols.^16^ Briefly, serum samples were tested for their capacity to neutralise MERS-CoV (EMC/2012 isolate) infections in vitro with 100 50% tissue culture infective doses in Huh-7 cells. Sera were incubated for 1 h with the virus and then added to the cells and incubated for 4 days. A recombinant human monoclonal antibody directed against the RBD of MERS-CoV S1 (MRO-895LC Creative Biolabs, Shirley, NY, USA) served as a neutralisation control. Full neutralisation was observed at a dilution of 1/512 (with or without one dilution step acceptable as inter-assay variation), which corresponds to a concentration of 0·2 μg/mL.^17^ Neutralisation titres were calculated as reciprocal values of geometric mean titres of four replicates. A titre of 8 was considered positive.

For the pseudovirus neutralisation assay, MERS-CoV EMC/2012, KOR/KNIH/002, and England-1 (endoplasmic reticulum retention signal-deleted, amino acids 1–1338) pseudotyped lentiviral particles were generated and titrated using lentivirus-associated p24 ELISA Kit (Cell Biolabs, San Diego, CA, USA).^18^ Two-fold diluted serum was incubated with 2 × 10^6^ viral particles of pseudotyped virus for 60 min at 37°C and added to 786-O cells, incubated for 6 h, and replaced with fresh medium; luciferase-reporter activity was measured 3 days later. A commercially available neutralising monoclonal antibody (40069-R723, Sino Biological, Beijing, China) was used as a positive control. All of the samples were analysed in duplicates. Data were expressed as geometric mean with SDs.

To assess cellular immunity, interferon-γ-linked enzyme-linked immunospot (ELISpot) assays were done with fresh peripheral blood mononuclear cells (PBMCs) to determine responses to the MERS-CoV spike vaccine antigen. Methodology was as described previously^19^ with the following exceptions. PBMCs were separated from whole blood within 4 h of venepuncture. 275 synthetic peptides (15mers overlapping by ten amino acids) spanning the entire vaccine insert, including the tissue plasminogen activator leader sequence, were used to stimulate PBMCs. Peptides were pooled into 13 pools for the MERS-CoV spike protein containing 18 or 21 peptides, plus a single pool of five peptides for the tissue plasminogen activator leader. Peptide sequences and pooling are summarised in the appendix (pp 4–7). Data were analysed according to a quality control standard operational procedure.

The lower limit of detection for the assay was 56 spot-forming cells (SFCs) for summed responses to the 13 MERS-CoV spike peptide pools.

### Outcomes

The primary outcomes were occurrence of solicited local reactogenicity signs and symptoms for 7 days after vaccination; occurrence of solicited systemic reactogenicity signs and symptoms for 7 days after vaccination; occurrence of unsolicited adverse events for 28 days after vaccination; change from day 0 (baseline) to day 28 for safety haematology (full blood count) and biochemistry (sodium, potassium, urea, creatinine, bilirubin, alanine aminotransferase, alkaline phosphatase, and albumin) laboratory measures; and occurrence of serious adverse events during the whole study duration of 12 months.

The secondary outcomes were cellular and humoral immunogenicity of ChAdOx1 MERS as measured by ELISpot, ELISA, and virus-neutralising antibody assays from baseline to 12 months.

### Statistical analysis

Safety endpoints are described as frequencies with their respective percentages alongside their 95% CIs. The association between the frequency of moderate or severe solicited adverse events and group allocation (intermediate dose and high dose) is reported as relative risk with the respective 95% CIs and p value (Fisher's exact test). Immunology data were tested for normal distribution using the D'Agostino-Pearson omnibus normality test. Data were analysed with non-parametric measures if data were not normally distributed or the sample size was small. Kruskal-Wallis with Dunn's multiple comparison post test was used to compare across timepoints or groups. p values of less than 0·05 were considered to be significant. For ELISpot data, values are SFCs per million PBMCs. Statistical analysis of safety and immunogenicity data was done with GraphPad Prism (version 8.01 for Windows).

This study is registered with ClinicalTrials.gov, NCT03399578.

### Role of the funding source

The funders of the study had no role in the study design, data collection, data analysis, data interpretation, or writing of the report. The corresponding author had full access to all the data in the study and had final responsibility for the decision to submit for publication.

## Results

**Figure 1:**
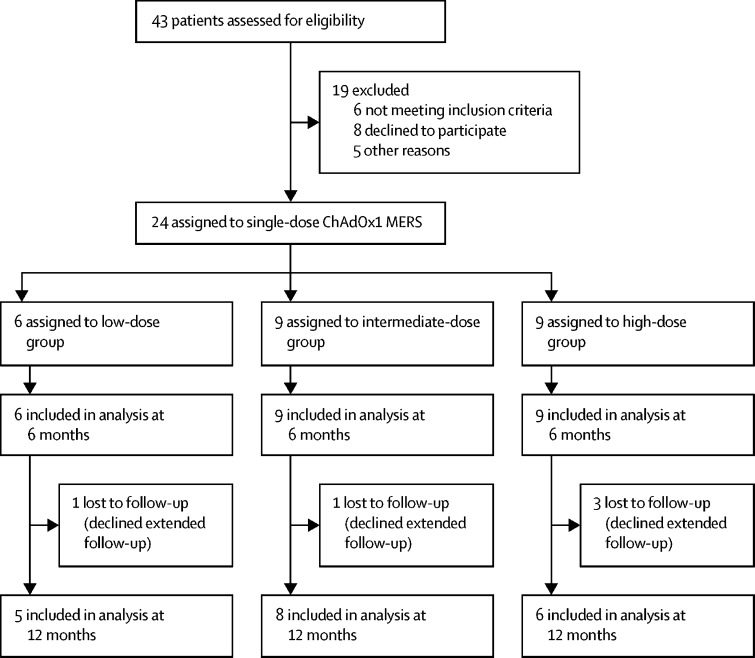
Trial profile MERS=Middle East respiratory syndrome.

Between March 14 and Aug 15, 2018, 24 healthy adults were enrolled: six were assigned to the low-dose group, nine to the intermediate-dose group, and nine to the high-dose group (figure 1; see table 1 for baseline characteristics). All participants received a single dose of ChAdOx1 MERS according to their group allocation. All participants were available for follow-up at 6 months, but five (one in the low-dose group, one in the intermediate-dose group, and three in the high-dose group) were lost to follow-up at 12 months (figure 1).

**Table 1:** Baseline characteristics

		**Low-dose group (n=6)**	**Intermediate-dose group (n=9)**	**High-dose group (n=9)**
Age, years		25·5 (19–37)	29 (23–43)	23 (20–47)
Sex				
	Male	2 (33%)	2 (22%)	3 (33%)
	Female	4 (67%)	7 (78%)	6 (67%)
Ethnicity				
	White	6 (100%)	9 (100%)	9 (100%)

Data are median (range) or n (%).

ChAdOx1 MERS was safe at doses up to 5 × 10^10^ viral particles with no vaccine-related serious adverse events reported during 12 months of follow-up. 124 local and systemic solicited adverse events were reported from vaccination to 7 days after vaccination (table 2). One serious adverse event reported was deemed to be not related to ChAdOx1 MERS. 92 (74% [95% CI 66–81]) solicited adverse events were mild, 31 (25% [18–33]) were moderate, and all were self-limiting. All solicited adverse events were completely resolved 6 days after vaccination and 119 (96%) had their onset within the first 72 h after vaccination (66 [53%] at day 0, 48 [39%] at day 1, and five [4%] at day 2). Injection site pain was the most common local adverse event, reported by 18 (75%) of 24 participants and was predominantly mild in severity (table 2). Fatigue was the most common systemic adverse event, followed by headache and malaise. Median duration of solicited adverse events is summarised in the appendix (p 1).

**Table 2:** Local and systemic solicited adverse events

	**Low-dose group (n=6)**			**Intermediate-dose group (n=9)**			**High-dose group (n=9)**		
	Mild	Moderate	Severe	Mild	Moderate	Severe	Mild	Moderate	Severe
Any symptom	5 (83%)	1 (17%)	0	5 (56%)	2 (22%)	0	0	8 (89%)	1 (11%)
Any local symptom	4 (67%)	0	0	7 (78%)	0	0	5 (56%)	4 (44%)	0
Injection site pain	2 (33%)	0	0	7 (78%)	0	0	5 (56%)	4 (44%)	0
Pruritus	2 (33%)	0	0	1 (11%)	0	0	2 (22%)	0	0
Warmth	1 (17%)	0	0	1 (11%)	0	0	2 (22%)	0	0
Swelling	0	0	0	0	0	0	0	0	0
Erythema	2 (33%)	0	0	1 (11%)	0	0	2 (22%)	0	0
Any systemic symptom	5 (83%)	1 (17%)	0	5 (56%)	2 (22%)	0	1 (11%)	7 (78%)	1 (11%)
Fever	0	0	0	1 (11%)	0	0	1 (11%)	3 (33%)	1 (11%)
Feverishness	1 (17%)	0	0	4 (44%)	0	0	4 (44%)	3 (33%)	0
Arthralgia	1 (17%)	0	0	2 (22%)	0	0	5 (56%)	0	0
Myalgia	2 (33%)	0	0	4 (44%)	0	0	5 (56%)	2 (22%)	0
Headache	3 (50%)	1 (17%)	0	5 (56%)	0	0	2 (22%)	5 (56%)	0
Fatigue	4 (67%)	0	0	5 (56%)	1 (11%)	0	3 (33%)	4 (44%)	0
Nausea	0	0	0	2 (22%)	1 (11%)	0	4 (44%)	1 (11%)	0
Malaise	1 (17%)	0	0	4 (44%)	1 (11%)	0	1 (11%)	5 (56%)	0

Six participants reported a short-lived fever, with a temperature higher than 37·5°C within the first 72 h after vaccination (one in the intermediate-dose group and five in the high-dose group). One participant in the high-dose group had a temperature of 39·6°C (classed as severe fever) on the day of vaccination as a result of the vaccination. This episode resolved within 24 h.

The proportion of moderate and severe adverse events was significantly higher in the high-dose group than in the intermediate-dose group (relative risk 5·83 [95% CI 2·11–17·42], p<0·0001), but there were no safety concerns despite higher reactogenicity.

Unsolicited adverse events in the 28 days following vaccination considered to be possibly, probably, or definitely related to ChAdOx1 MERS were predominantly mild in nature and resolved within the follow-up period of 12 months (appendix p 2). Laboratory adverse events considered to be at least possibly related to the study intervention were self-limiting and predominantly mild in severity (appendix p 3).

**Figure 2:**
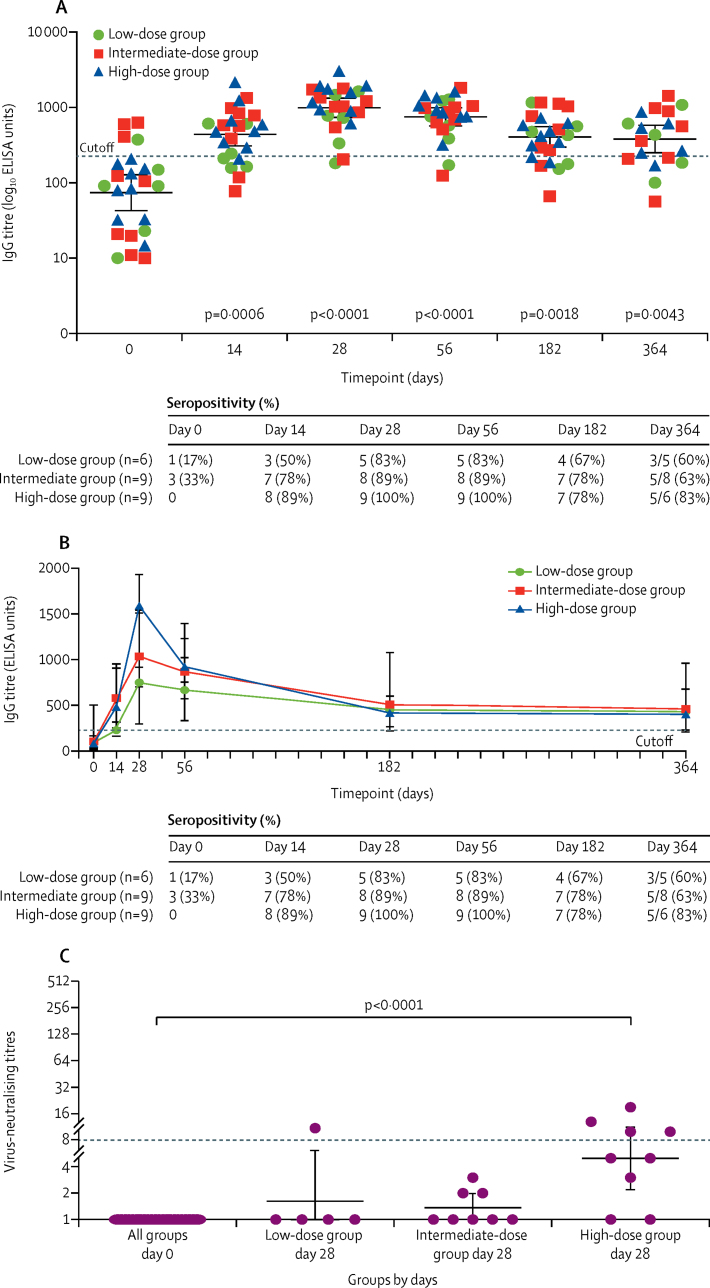
Humoral responses to ChAdOx1 MERS vaccine (A) Individual IgG titres at each dose group. Data points represent geometric means, and error bars represent 95% CIs. The dashed line represents the cutoff value for seropositivity. (B) Data points represent median and error bars represent IQRs for IgG titres in each group. The dashed line represents the cutoff value for seropositivity (225 ELISA units). (C) Virus-neutralising titres at day 0 and day 28 for each group. Data points represent geometric means, and error bars represent 95% CIs. The dashed line represents the lower limit of detection under our experimental condition. p values calculated by Kruskal-Wallis with Dunn's multiple comparison post test. MERS=Middle East respiratory syndrome.

A single dose of the vaccine induced a strong antibody response in 22 (92%) participants across all groups, which persisted for 1 year after vaccination in participants who were not lost to follow-up. Antibody responses increased rapidly after vaccination, peaking 28 days after vaccination for all groups (figure 2A). Seroconversion occurred in 18 (75%) of 24 participants at 14 days after vaccination, increasing to 22 (92%) participants up to 56 days after vaccination. Seropositivity was maintained in 13 (68%) of 19 participants up to 1 year after vaccination (geometric mean 381·1 ELISA units [95% CI 251·2–578·3], p=0·0043, day 0 *vs* day 364). Antibody responses peaked at day 28, with the high-dose group producing the highest antibody response (figure 2B). IgG titres increased significantly at day 28 and day 56 after vaccination with increasing ChAdOx1 MERS dose compared with baseline (figure 2B; appendix p 8), and no significant differences in anti-MERS IgG was found between the dose groups at these timepoints (data not shown). Detectable anti-MERS IgG titres were observed in four (17%) of 24 participants at baseline. However, this baseline response did not inhibit the antibody responses to the vaccine, as evident by the increased IgG titres observed for these participants at day 28 and day 56 after vaccination (appendix p 9).

Neutralising antibodies against live MERS-CoV were measured from 22 participants (five in the low-dose group, eight in the intermediate-dose group, and nine in the high-dose group). Virus neutralising antibody titres were measured at day 0 and were negative, and at day 28. Neutralising antibodies were detected only in the low-dose group (one of five participants) and high-dose group (four [44%, 95% CI 19–73] of nine participants), with only the high-dose group producing a significant increase in neutralising antibody titres compared with the baseline (p<0·0001, Kruskall-Wallis with Dunn's multiple comparisons test; figure 2C) in an assay testing neutralisation of live MERS-CoV heterologous to the vaccine-derived strain. A further exploratory investigation of the association between the total IgG and neutralising antibody titres for the high-dose group at the peak antibody response showed a moderate positive correlation between the total IgG and neutralising antibody titres (appendix p 10).

**Figure 3:**
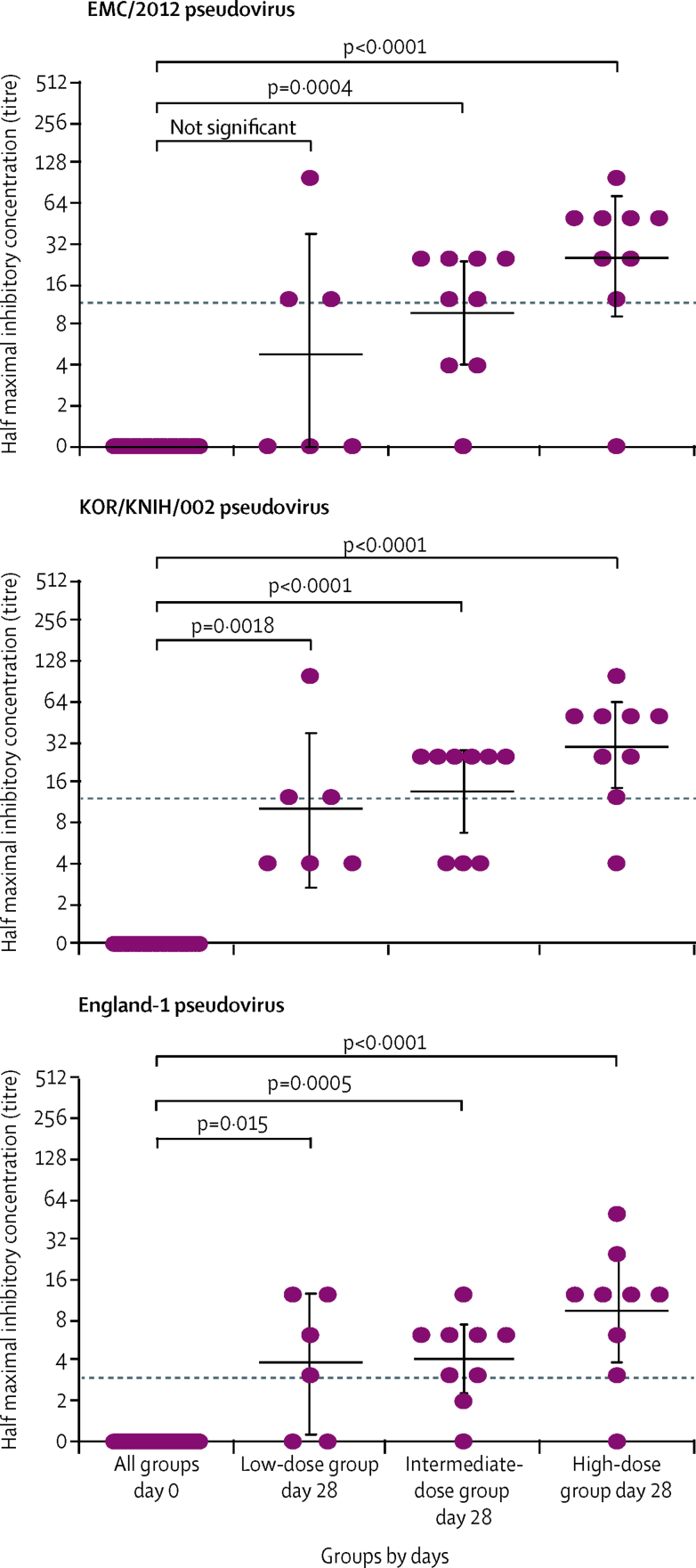
MERS-CoV spike-pseudotyped neutralisation p values were calculated using Kruskall-Wallis with Dunn's multiple comparison post test. The dashed lines represent lower limit of detection under our experimental condition. Data points represent geometric means, and error bars represent 95% CIs. MERS-CoV=Middle East respiratory syndrome coronavirus.

Neutralisation tests of three pseudotyped lentiviruses expressing spike glycoprotein from three different MERS-CoV strains were negative at day 0, but 17 (71% [95% CI 49–87]) of 24 samples were positive at day 28 when tested against EMC/2012 and KOR/KNIH/002, and 19 (79% [95% CI 58–93]) were positive when tested against England-1 at day 28 (figure 3). The neutralising antibody titres measured against the live MERS virus for the high-dose group correlated significantly with those measured using the three different pseudotyped lentiviral particles (appendix p 11).

Cellular immunogenicity to ChAdOx1 MERS was assessed by ex-vivo ELISpot. Responses to the negative control (PBMCs with no stimulation) were low, with a median of 17·3 SFCs (IQR 9·3–22·7). As expected, no response to the exogenous tissue plasminogen leader sequence was detected (median 5 SFCs [IQR 4–12]; data not shown).

**Figure 4:**
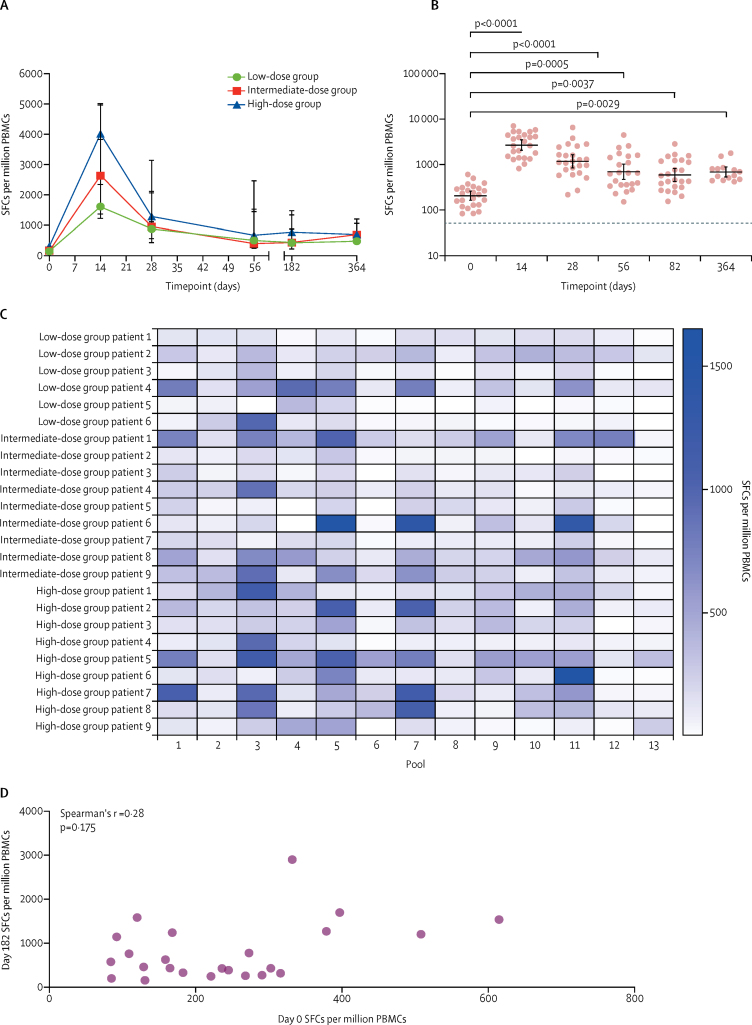
T-cell responses to ChAdOx1 MERS Ex-vivo interferon-γ-linked enzyme-linked immunospot responses to MERS spike protein. Panels A, B, and D show the total response to MERS spike peptides (sum of 13 pools), and panel C shows the response to each pool. In the low-dose group, data were missing for three participants for day 364. In the intermediate group, data were missing for one participant for day 56 and three participants for day 364. In the high-dose group, data were missing for four participants for day 364. (A) Data points represent median and error bars represent IQR for SFCs per million PBMCs during 12-month follow-up. (B) Responses for individual participants. Data points represent geometric means, and error bars represent 95% CIs. The dashed line represents the lower limit of detection under our experimental condition. Kruskal-Wallis with Dunn's multiple comparison post test. (C) Heat map of responses to each peptide pool for each participant, arranged by ascending dose group. (D) Correlation between baseline (day 0) and 6-month (day 182) responses. MERS=Middle East respiratory syndrome. PBMCs=peripheral blood mononuclear cells. SFCs=spot-forming cells.

High frequencies of interferon-γ-secreting T cells recognising MERS spike peptides were elicited (figure 4). Data from six (4%) of 139 assays were removed from the final dataset because negative controls were above the predefined quality control parameters. Responses peaked at 14 days after vaccination for all groups (median 1617 SFCs [IQR 1227–3833] for the low-dose group, 2631 SFCs [1373–4966] for the intermediate-dose group, and 4019 SFCs [2349–5013] for the high-dose group; figure 4A). No significant effect of vaccine dose on magnitude of response was detected at any timepoint; therefore, data were pooled for subsequent analyses. We observed a significant increase in T-cell response compared with baseline at all timepoints (figure 4B). Of note, this increase persisted to 1 year after vaccination at four times higher than baseline (geometric mean 695·6 SFCs [95% CI 537·5–900·2], p=0·0029, day 364 *vs* day 0). Individual T-cell responses per dose group are shown in the appendix (p 12).

We investigated the relative contribution of individual peptide pools at the peak timepoint (day 14) to identify regions of immunodominance within the antigen (figure 4C). Pools 3, 5, 7, and 11 were the most frequently recognised. Responses to the S1 subunit were higher than for S2 at day 14 for most participants (appendix p 13). T-cell responses to both the RBD (within S1) and S2 persisted to day 364 after vaccination (appendix p 13).

We detected responses to MERS spike peptides before vaccination (geometric mean 208·9 SFCs [95% CI 164·9–264·6]; figure 4B; appendix p 12) in four participants (one in the low-dose group and three in the intermediate-dose group). These were mainly towards the RBD peptides (pools 4–6; appendix p 13). No correlation was found between the magnitude of pre-existing and post-vaccination responses at day 182 (Spearman's r=0·28, p=0·175; figure 4D). No association between day 0 responses and HLA type was detected (data not shown), and there was no association between the magnitude of pre-existing T-cell immunity and humoral responses measured at day 0. There was no association between the magnitude of T-cell and antibody responses to ChAdOx1 MERS at day 28 (Spearman's r=–0·05, p=0·82; appendix p 14).

## Discussion

In this study, we have shown that the candidate ChAdOx1 MERS vaccine given as a single dose was safe and well tolerated in all three groups, although a higher reactogenicity profile was observed at the dose of 5 × 10^10^ viral particles, with five of nine participants in that group reporting short-lived fever (temperature higher than 37·5°C). No serious adverse reactions occurred. The majority of adverse events reported were mild or moderate in severity, and all adverse events were self-limiting. The profile of adverse events reported in this trial is similar to that for another ChAdOx1 vectored vaccine expressing influenza A antigens and other closely related simian adenoviruses, such as ChAdOx2, ChAd3, and ChAd63 vectored vaccines expressing different antigens.^19^, ^20^, ^21^, ^22^, ^23^

Safety concerns around the use of full-length coronavirus spike glycoproteins as a vaccine antigen have been raised following historical reports of immunopathology and antibody-dependent enhancement reported in vitro and post-SARS-CoV challenge in mice, ferrets, and non-human primates immunised with whole SARS-CoV-inactivated or full-length spike protein-based vaccines.^24^, ^25^, ^26^ So far, one study has reported lung immunopathology following MERS-CoV challenge in mice immunised with an inactivated MERS-CoV candidate vaccine.^27^ However, in preclinical studies of ChAdOx1 immunisation and MERS-CoV challenge, no antibody-dependent enhancement was observed in transgenic mice expressing human DPP4, dromedary camels, or non-human primates.^14^, ^15^, ^28^

The vaccine was immunogenic at all doses, inducing seroconversion in the majority of participants and T-cell responses in all, with responses demonstrating good durability up to 1 year after vaccination. Onset of detectable immune responses was rapid, with T-cell responses peaking 14 days after vaccination and antibodies at 28 days. A small number of participants had positive antibody or T-cell responses (but never both) before vaccination. These pre-exisiting responses are likely to be due to cross-reactivity with other known human coronaviruses, such as HCoV-229E, HCoV-HKU1, HCoV-NL63, and HCoV-OC43, all of which circulate worldwide and can cause lower respiratory tract infections.^9^ The prevalence of seropositivity to these four viruses in the UK is unknown, but a cross-sectional survey in the Netherlands found that 100% of children had seroconverted to at least one of these by the age of 10 years, and it is likely that the rate is similar in the UK.^29^ With the emergence of SARS-CoV-2, pre-existing cross-reactive immune responses to human coronaviruses is likely to be an area of further investigation. Importantly, pre-existing T-cell or antibody responses did not affect vaccine immunogenicity and no neutralising antibodies were detected before vaccination.

Correlates of protection for MERS-CoV are currently unknown. Neutralising antibodies targeting different epitopes of the spike glycoprotein have been associated with protection against MERS-CoV challenge in animal models.^30^ Here, we demonstrated that a single dose of ChAdOx1 MERS was able to elicit neutralising antibodies against live MERS-CoV in 44% of participants receiving the high dose. Between 71% and 79% of all participants produced neutralising antibodies in assays using pseudotyped lentiviruses expressing the spike glycoprotein from three different strains of MERS-CoV. Despite differences in methodology, there was a strong positive correlation between neutralising antibodies from pseudotyped viruses and neutralising antibodies obtained from the live virus assay in the high-dose group. Strategies to increase neutralising antibody seroconversion include a two-dose regimen, and investigations for this hypothesis are underway. Importantly, in MERS survivors CD8 T-cell responses were found to correlate with less severe disease and lower virus shedding.^31^ ChAdOx1 MERS vaccination resulted in significant increases in MERS spike-specific T-cell responses that were maintained for at least a year after vaccination at four times higher than baseline. Adenoviral vectored vaccines are potent inducers of CD8 T-cell responses, and the phenotype of T cells induced by vaccination will be determined in further studies.

MERS-CoV vaccines are required for both camels and humans. ChAdOx1 MERS has been tested in dromedary camels in Saudi Arabia and was shown to significantly reduce viral shedding, which could potentially translate into reduced zoonotic transmission.^15^ The One Health vaccine development approach used here, by which the same vaccine is co-developed for humans and susceptible animal species, allows vaccine efficacy to be tested in an appropriate animal model, which could support licensure of the vaccine for humans. Target groups include people who are occupationally exposed to camels and health-care workers. However, severe and fatal cases of MERS-CoV disproportionally affect individuals older than 50 years and those with comorbidities. Therefore, it is paramount that vaccines developed against MERS-CoV are suitable for administration in older age groups in the context of an outbreak. The use of replication-deficient vectors avoids the risks of inadequate attenuation of replication-competent vaccines, which could potentially lead to disseminated disease in immunocompromised hosts. Immunogenicity of a ChAdOx1 vectored vaccine against influenza has been demonstrated in older adults (50–78 years of age).^20^ For use in outbreaks, rapid onset of immunity after a single dose, as demonstrated here, is highly desirable.

The Coalition for Epidemic Preparedness Innovations is supporting the clinical development of four novel vaccines against MERS, all of which express the full-length spike glycoprotein. In addition to ChAdOx1 MERS, a DNA vaccine and two other viral vectored vaccines (MVA and measles vectors) are being developed.^16^, ^32^, ^33^ Data from early clinical trials will support the use of one or more of these vaccines in the Middle East or as a stockpile suitable for outbreak response in any country.

Limitations of this study include the small sample size and the open-label, non-randomised, and uncontrolled trial design. Long-term safety and immunogenicity findings should be interpreted with caution considering that five of 24 participants declined the 12-month extended follow-up. Further research is required to better understand the importance of pre-existing immunity and cross-reactivity to other coronaviruses in the context of emerging coronaviruses outbreaks. Nonetheless, this study provides valuable information on reactogenicity and immunogenicity of the first clinical use of ChAdOx1 MERS.

In conclusion, ChAdOx1 MERS was safe and well tolerated at all tested doses. A single dose was able to elicit both humoral and cellular responses against MERS-CoV. The results of this first-in-human clinical trial support clinical development progression into phase 1b and 2 trials in the Middle East. Healthy adults, health-care workers, people who are occupationally exposed to camels, and older age groups with comorbidities will be recruited and assessed for safety and immunogenicity of ChAdOx1 MERS to be given as a single or two-dose administration regimen.

**This online publication has been corrected. The first corrected version first appeared at thelancet.com/infection on May 12, 2020 and the second on June 8, 2020**

## Data sharing

The study protocol is available with this publication as part of the supplementary material. Individual participant data are available upon request directed to the corresponding author and after approval of a proposal can be shared through a secure online platform.
