# Supplementary material for: Safety and immunogenicity of a candidate Middle East respiratory syndrome coronavirus viral-vectored vaccine: a dose-escalation, open-label, non-randomised, uncontrolled, phase 1 trial
Source: Lancet Infect Dis. 2020 Jul;20(7):816–26. doi: 10.1016/S1473-3099(20)30160-2 (PMC7172901; doi:10.1016/S1473-3099(20)30160-2)
Supplement: Supplementary appendix [file mmc1.pdf]

THE LANCET Infectious Diseases

Supplementary webappendix

This webappendix formed part of the original submission and has been peer reviewed. We post it as supplied by the authors.

Supplement to: Folegatti PM, Bittaye M, Flaxman A, et al. Safety and immunogenicity of a candidate Middle East respiratory syndrome coronavirus viral-vectored vaccine: a dose-escalation, open-label, non-randomised, uncontrolled, phase 1 trial. *Lancet Infect Dis* 2020; published online April 20. https://doi.org/10.1016/S1473-3099(20)30160-2.

Supplementary Material

Supplementary Table 1. Summary of median duration in days of reported solicited AEs (all groups).

AE	Median	IQR*	Maximum duration
**Pain at injection site**	3.5	1-4.25	6
**Pruritus**	1	1-1	1
**Warmth**	1	1-1.75	2
**Erythema**	1	1-1	1
**Fever**	1	1-1	1
**Feverishness**	1.5	1-2	3
**Arthralgia**	1	1-2	4
**Myalgia**	1	1-2.5	4
**Headache**	2	1-2	5
**Fatigue**	2	1-3	4
**Nausea**	1	1-3.75	5
**Malaise**	1	1-2	3
**Overall**	1	1-2	6

*Interquartile range

Supplementary Table 2. Unsolicited adverse events considered possibly, probably or definitely related to ChAdOx1 MERS

Subject ID	MEDDRA PT	MEDDRA PT CODE	Day of Onset	Duration (days)	Max. Severity
00101006	Nasal congestion	10028735	1	10	1
00101006	Rhinorrhoea	10039101	1	8	1
00101006	Diarrhoea	10012735	5	1	1
00101006	Nasopharyngitis	10028810	10	1	1
00101011	Back pain	10003988	0	2	1
00101011	Lymphadenopathy	10025197	1	6	1
00101011	Odynophagia	10030094	1	6	1
00101039	Orthostatic hypotension	10031127	0	1	2
00101012	Photophobia	10034960	1	1	1
00101012	Ear pain	10014020	1	1	1
00101012	Arthralgia	10003239	1	1	1
00101012	Arthralgia	10003239	1	5	1
00101010	Decreased appetite	10061428	1	1	1
00101010	Diarrhoea	10012735	2	1	2
00101010	Vomiting	10047700	4	2	2
00101013	Dizziness	10013573	2	1	1
00101027	Dizziness	10013573	0	1	1
00101027	Back pain	10003988	0	2	1
00101027	Neck pain	10028836	1	1	1
00101027	Abdominal pain upper	10000087	1	1	1
00101017	Neck pain	10028836	1	1	1
00101015	Feeling cold	10016326	0	1	1
00101015	Rash	10037844	1	2	1
00101020	Nausea	10028813	7	2	1
00101019	Productive cough	10036790	1	1	1
00101019	Sneezing	10041232	1	1	1
00101019	Ear pain	10014020	6	2	1
00101032	Chills	10008531	0	1	2
00101032	Feeling cold	10016326	0	1	2
00101032	Decreased appetite	10061428	1	1	1
00101032	Alopecia	10001760	7	50	2
00101037	Chills	10008531	0	1	1
00101037	Infrequent bowel movements	10059158	2	1	1
00101037	Headache	10019211	13	1	1
00101033	Headache	10019211	7	2	1
00101038	Chills	10008531	0	1	2
00101038	Eczema	10014184	26	1	1
00101038	Pityriasis rosea	10035114	26	56	3

Supplementary Table 3. Laboratory AEs considered possibly, probably or definitively related to ChAdOx1 MERS

ID	Group	Event	Severity	Onset	Resolved by
101013	2	Neutropenia	Mild	D7	D28
101014	3	Neutropenia	Moderate	D2	D7
101015	3	Leucopoenia	Mild	D2	D7
101015	3	Lymphopenia	Mild	D2	D7
101020	3	Leucopoenia	Mild	D2	D7
101020	3	Neutropenia	Mild	D2	D7
101024	2	Leucopoenia	Mild	D2	D7
101024	2	Neutropenia	Mild	D2	D7
101032	3	Neutropenia	Mild	D2	D7
101035	2	Neutropenia	Mild	D2	D7
101038	3	Neutropenia	Mild	D2	D7
101039	1	Anaemia	Mild	D7	D28

Supplementary Table 4. Peptide pools for ELISpot analysis

Pool	Peptide no	Sequence	Pool	Peptide no	Sequence
tPA	tPA1	MDAMKRGLCCVLLLC	Pool 8	MERS148	PLGQSLCALPDTPST
	tPA2	RGLCCVLLLCGAVFV		MERS149	LCALPDTPSTLTPRS
	tPA3	VLLLCGAVFVSASQE		MERS150	DTPSTLTPRSVRSVP
	tPA4	GAVFVSASQEIHARF		MERS151	LTPRSVRSVPGEMRL
	tPA5	SASQEIHARFRRIHS		MERS152	VRSVPGEMRLASIAF
Pool 1	MERS1	IHARFRRIHSVFLLM		MERS153	GEMRLASIAFNHPIQ
	MERS2	RRIHSVFLLMFLLTP		MERS154	ASIAFNHPIQVDQLN
	MERS3	VFLLMFLLTPTESYV		MERS155	NHPIQVDQLNSSYFK
	MERS4	FLLTPTESYVDVGPD		MERS156	VDQLNSSYFKLSIPT
	MERS5	TESYVDVGPDSVKSA		MERS157	SSYFKLSIPTNFSFG
	MERS6	DVGPDSVKSACIEVD		MERS158	LSIPTNFSFGVTQEY
	MERS7	SVKSACIEVDIQQTF		MERS159	NFSFGVTQEYIQTTI
	MERS8	CIEVDIQQTFFDKTW		MERS160	VTQEYIQTTIQKVTV
	MERS9	IQQTFFDKTWPRPID		MERS161	IQTTIQKVTVDCKQY
	MERS10	FDKTWPRPIDVSKAD		MERS162	QKVTVDCKQYVCNGF
	MERS11	PRPIDVSKADGIIYP		MERS163	DCKQYVCNGFQKCEQ
	MERS12	VSKADGIIYPQGRTY		MERS164	VCNGFQKCEQLLREY
	MERS13	GIIYPQGRTYSNITI		MERS165	QKCEQLLREYGQFCS
	MERS14	QGRTYSNITITYQGL		MERS166	LLREYGQFCSKINQA
	MERS15	SNITITYQGLFPYQG		MERS167	GQFCSKINQALHGAN
	MERS16	TYQGLFPYQGDHGDM		MERS168	KINQALHGANLRQDD
	MERS17	FPYQGDHGDMYVYSA	Pool 9	MERS169	LHGANLRQDDSVRNL
	MERS18	DHGDMYVYSAGHATG		MERS170	LRQDDSVRNLFASVK
	MERS19	YVYSAGHATGTTPQK		MERS171	SVRNLFASVKSSQSS
	MERS20	GHATGTTPQKLFVAN		MERS172	FASVKSSQSSPIIPG
	MERS21	TTPQKLFVANYSQDV		MERS173	SSQSSPIIPGFGGDF
Pool 2	MERS22	LFVANYSQDVKQFAN		MERS174	PIIPGFGGDFNLTLL
	MERS23	YSQDVKQFANGFVVR		MERS175	FGGDFNLTLLEPVSI
	MERS24	KQFANGFVVRIGAAA		MERS176	NLTLLEPVSISTGSR
	MERS25	GFVVRIGAAANSTGT		MERS177	EPVSISTGSRSARSA
	MERS26	IGAAANSTGTVIISP		MERS178	STGSRSARSAIEDLL
	MERS27	NSTGTVIISPSTSAT		MERS179	SARSAIEDLLFDKVT
	MERS28	VIISPSTSATIRKIY		MERS180	IEDLLFDKVTIADPG
	MERS29	STSATIRKIYPAFML		MERS181	FDKVTIADPGYMQGY
	MERS30	IRKIYPAFMLGSSVG		MERS182	IADPGYMQGYDDCMQ
	MERS31	PAFMLGSSVGNFSDG		MERS183	YMQGYDDCMQQGPAS
	MERS32	GSSVGNFSDGKMGRF		MERS184	DDCMQQGPASARDLI
	MERS33	NFSDGKMGRFFNHTL		MERS185	QGPASARDLICAQYV
	MERS34	KMGRFFNHTLVLLPD		MERS186	ARDLICAQYVAGYKV
	MERS35	FNHTLVLLPDGCGTL		MERS187	CAQYVAGYKVLPPLM
	MERS36	VLLPDGCGTLLRAFY		MERS188	AGYKVLPPLMDVNME
	MERS37	GCGTLLRAFYCILEP		MERS189	LPPLMDVNMEAAYTS
	MERS38	LRAFYCILEPRSGNH	Pool 10	MERS190	DVNMEAAYTSSLLGS
	MERS39	CILEPRSGNHCPAGN		MERS191	AAYTSSLLGSIAGVG
	MERS40	RSGNHCPAGNSHTSF		MERS192	SLLGSIAGVGWTAGL
	MERS41	CPAGNSHTSFATYHT		MERS193	IAGVGWTAGLSSFAA
	MERS42	SHTSFATYHTPATDC		MERS194	WTAGLSSFAAIPFAQ
Pool 3	MERS43	ATYHTPATDCSDGNY		MERS195	SSFAAIPFAQSIFYR
	MERS44	PATDCSDGNYNRNAS		MERS196	IPFAQSIFYRLNGVG
	MERS45	SDGNYNRNASLNSFK		MERS197	SIFYRLNGVGITQQV
	MERS46	NRNASLNSFKEYFNL		MERS198	LNGVGITQQVLSENQ
	MERS47	LNSFKEYFNLRNCTF		MERS199	ITQQVLSENQKLIAN
	MERS48	EYFNLRNCTFMYTYN		MERS200	LSENQKLIANKFNQA
	MERS49	RNCTFMYTYNITEDE		MERS201	KLIANKFNQALGAMQ
	MERS50	MYTYNITEDEILEWF		MERS202	KFNQALGAMQTGFTT

	MERS51	ITEDEILEWFGITQT		MERS203	LGAMQTGFTTTNEAF
	MERS52	ILEWFGITQTAQGVH		MERS204	TGFTTTNEAFRKVQD
	MERS53	GITQTAQGVHLFSSR		MERS205	TNEAFRKVQDAVNNN
	MERS54	AQGVHLFSSRYVDLY		MERS206	RKVQDAVNNNAQALS
	MERS55	LFSSRYVDLYGGNMF		MERS207	AVNNNAQALSKLASE
	MERS56	YVDLYGGNMFQFATL		MERS208	AQALSKLASELSNTF
	MERS57	GGNMFQFATLPVYDT		MERS209	KLASELSNTFGAISA
	MERS58	QFATLPVYDTIKYYS		MERS210	LSNTFGAISASIGDI
	MERS59	PVYDTIKYYSIIPHS	Pool 11	MERS211	GAISASIGDIIQRLD
	MERS60	IKYYSIIPHSIRSIQ		MERS212	SIGDIIQRLDVLEQD
	MERS61	IIPHSIRSIQSDRKA		MERS213	IQRLDVLEQDAQIDR
	MERS62	IRSIQSDRKAWAAFY		MERS214	VLEQDAQIDRLINGR
	MERS43	ATYHTPATDCSDGNY		MERS215	AQIDRLINGRLTTLN
	MERS44	PATDCSDGNYNRNAS		MERS216	LINGRLTTLNAFVAQ
	MERS45	SDGNYNRNASLNSFK		MERS217	LTTLNAFVAQQLVRS
	MERS46	NRNASLNSFKEYFNL		MERS218	AFVAQQLVRSESAAL
	MERS47	LNSFKEYFNLRNCTF		MERS219	QLVRSESAALSAQLA
	MERS48	EYFNLRNCTFMYTYN		MERS220	ESAALSAQLAKDKVN
	MERS49	RNCTFMYTYNITEDE		MERS221	SAQLAKDKVNECVKA
	MERS50	MYTYNITEDEILEWF		MERS222	KDKVNECVKAQSKRS
	MERS51	ITEDEILEWFGITQT		MERS223	ECVKAQSKRSGFCGQ
	MERS52	ILEWFGITQTAQGVH		MERS224	QSKRSGFCGQGTHIV
	MERS53	GITQTAQGVHLFSSR		MERS225	GFCGQGTHIVSFVVN
	MERS54	AQGVHLFSSRYVDLY		MERS226	GTHIVSFVVNAPNGL
	MERS55	LFSSRYVDLYGGNMF		MERS227	SFVVNAPNGLYFMHV
	MERS56	YVDLYGGNMFQFATL		MERS228	APNGLYFMHVGYYPS
	MERS57	GGNMFQFATLPVYDT		MERS229	YFMHVGYYPSNHIEV
	MERS58	QFATLPVYDTIKYYS		MERS230	GYYPSNHIEVVSAYG
	MERS59	PVYDTIKYYSIIPHS		MERS231	NHIEVVSAYGLCDAA
	MERS60	IKYYSIIPHSIRSIQ	Pool 12	MERS232	VSAYGLCDAANPTNC
	MERS61	IIPHSIRSIQSDRKA		MERS233	LCDAANPTNCIAPVN
	MERS62	IRSIQSDRKAWAAFY		MERS234	NPTNCIAPVNGYFIK
Pool4	MERS64	WAAFYVYKLQPLTFL		MERS235	IAPVNGYFIKTNNTR
	MERS65	VYKLQPLTFLLDFSV		MERS236	GYFIKTNNTRIVDEW
	MERS66	PLTFLLDFSVDGYIR		MERS237	TNNTRIVDEWSYTGS
	MERS67	LDFSVDGYIRRAIDC		MERS238	IVDEWSYTGSSFYAP
	MERS68	DGYIRRAIDCGFNDL		MERS239	SYTGSSFYAPEPITS
	MERS69	RAIDCGFNDLSQLHC		MERS240	SFYAPEPITSLNTKY
	MERS70	GFNDLSQLHCSYESF		MERS241	EPITSLNTKYVAPQV
	MERS71	SQLHCSYESFDVESG		MERS242	LNTKYVAPQVTYQNI
	MERS72	SYESFDVESGVYSVS		MERS243	VAPQVTYQNISTNLP
	MERS73	DVESGVYSVSSFEAK		MERS244	TYQNISTNLPPPLLG
	MERS74	VYSVSSFEAKPSGSV		MERS245	STNLPPPLLGNSTGI
	MERS75	SFEAKPSGSVVEQAE		MERS246	PPLLGNSTGIDFQDE
	MERS76	PSGSVVEQAEGVECD		MERS247	NSTGIDFQDELDEFF
	MERS77	VEQAEGVECDFSPLL		MERS248	DFQDELDEFFKNVST
	MERS78	GVECDFSPLLSGTPP		MERS249	LDEFFKNVSTSIPNF
	MERS79	FSPLLSGTPPQVYNF		MERS250	KNVSTSIPNFGSLTQ
	MERS80	SGTPPQVYNFKRLVF		MERS251	SIPNFGSLTQINTTL
	MERS81	QVYNFKRLVFTNCNY		MERS252	GSLTQINTTLLDLTY
	MERS82	KRLVFTNCNYNLTKL	Pool 13	MERS253	INTTLLDLTYEMLSL
	MERS83	TNCNYNLTKLLSLFS		MERS254	LDLTYEMLSLQQVVK
	MERS84	NLTKLLSLFSVNDFT		MERS255	EMLSLQQVVKALNES
Pool 5	MERS85	LSLFSVNDFTCSQIS		MERS256	QQVVKALNESYIDLK
	MERS86	VNDFTCSQISPAAIA		MERS257	ALNESYIDLKELGNY
	MERS87	CSQISPAAIASNCYS		MERS258	YIDLKELGNYTYYNK
	MERS88	PAAIASNCYSSLILD		MERS259	ELGNYTYYNKWPWYI
	MERS89	SNCYSSLILDYFSYP		MERS260	TYYNKWPWYIWLGFI

	MERS90	SLILDYFSYPLSMKS		MERS261	WPWYIWLGFIAGLVA
	MERS91	YFSYPLSMKSDLSVS		MERS262	WLGFIAGLVALALCV
	MERS92	LSMKSDLSVSSAGPI		MERS263	AGLVALALCVFFILC
	MERS93	DLSVSSAGPISQFNY		MERS264	LALCVFFILCCTGCG
	MERS94	SAGPISQFNYKQSFS		MERS265	FFILCCTGCGTNCMG
	MERS95	SQFNYKQSFSNPTCL		MERS266	CTGCGTNCMGKLKCN
	MERS96	KQSFSNPTCLILATV		MERS267	TNCMGKLKCNRCCDR
	MERS97	NPTCLILATVPHNLT		MERS268	KLKCNRCCDRYEEYD
	MERS98	ILATVPHNLTTITKP		MERS269	RCCDRYEEYDLEPHK
	MERS99	PHNLTTITKPLKYSY		MERS270	YEEYDLEPHKVHVH
	MERS100	TITKPLKYSYINKCS			
	MERS101	LKYSYINKCSRLLSD			
	MERS102	INKCSRLLSDDRTEV			
	MERS103	RLLSDDRTEVPQLVN			
	MERS104	DRTEVPQLVNANQYS			
	MERS105	PQLVNANQYSPCVSI			
Pool 6	MERS106	ANQYSPCVSIVPSTV			
	MERS107	PCVSIVPSTVWEDGD			
	MERS108	VPSTVWEDGDYYRKQ			
	MERS109	WEDGDYYRKQLSPLE			
	MERS110	YYRKQLSPLEGGGWL			
	MERS111	LSPLEGGGWLVASGS			
	MERS112	GGGWLVASGSTVAMT			
	MERS113	VASGSTVAMTEQLQM			
	MERS114	TVAMTEQLQMGFGIT			
	MERS115	EQLQMGFGITVQYGT			
	MERS116	GFGITVQYGTDTNSV			
	MERS117	VQYGTDTNSVCPKLE			
	MERS118	DTNSVCPKLEFANDT			
	MERS119	CPKLEFANDTKIASQ			
	MERS120	FANDTKIASQLGNCV			
	MERS121	KIASQLGNCVEYSLY			
	MERS122	LGNCVEYSLYGVSGR			
	MERS123	EYSLYGVSGRGVFQN			
	MERS124	GVSGRGVFQNCTAVG			
	MERS125	GVFQNCTAVGVRQQR			
	MERS126	CTAVGVRQQRFVYDA			
Pool 7	MERS127	VRQQRFVYDAYQNLV			
	MERS128	FVYDAYQNLVGYYSD			
	MERS129	YQNLVGYYSDDGNYY			
	MERS130	GYYSDDGNYYCLRAC			
	MERS131	DGNYYCLRACVSVPV			
	MERS132	CLRACVSVPVSVIYD			
	MERS133	VSVPVSVIYDKETKT			
	MERS134	SVIYDKETKTHATLF			
	MERS135	KETKTHATLFGSVAC			
	MERS136	HATLFGSVACEHISS			
	MERS137	GSVACEHISSTMSQY			
	MERS138	EHISSTMSQYSRSTR			
	MERS139	TMSQYSRSTRSMLKR			
	MERS140	SRSTRSMLKRRDSTY			

	MERS141	SMLKRRDSTYGPLQT			
	MERS142	RDSTYGPLQTPVGCV			
	MERS143	GPLQTPVGCVLGLVN			
	MERS144	PVGCVLGLVNSSLFV			
	MERS145	LGLVNSSLFVEDCKL			
	MERS146	SSLFVEDCKLPLGQS			
	MERS147	EDCKLPLGQSLCALP			

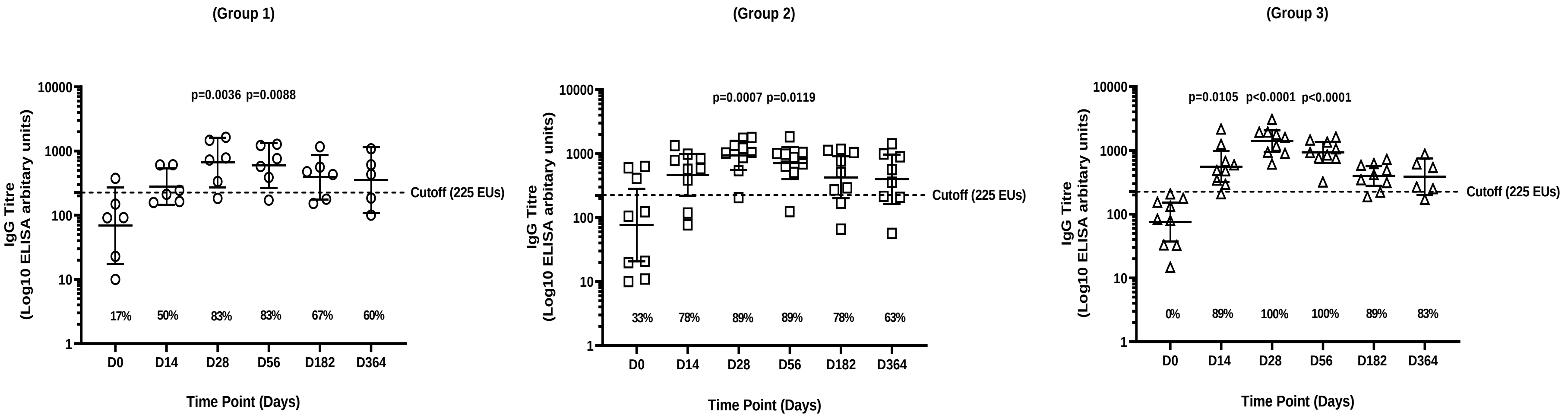

Supplementary Figure 1A: Antibody responses to ChAdOx1 MERS by dose group. Individual antibody responses for the three dose groups (Group 1, Group 2 and Group 3) at all timepoints are shown. The error bars represent geometric mean with 95% confidence interval and statistical significance by Kruskal-Wallis with Dunn's multiple comparison post-test is shown for each dose group. The percentage seropositivity at each timepoint for the three dose groups are shown in each individual graph. A cut-off value of 225 EUs was used to determine seropositivity and this was calculated using the mean plus three standard deviations of the EU values of 40 MERS negative pre-vaccinated volunteer plasma samples.

Group 1, n=6 volunteers for all time points except D364 where n=5
Group 2, n=9 volunteers for all time points except D364 where n=8
Group 3, n=9 volunteers for all time points except D364 where n=6

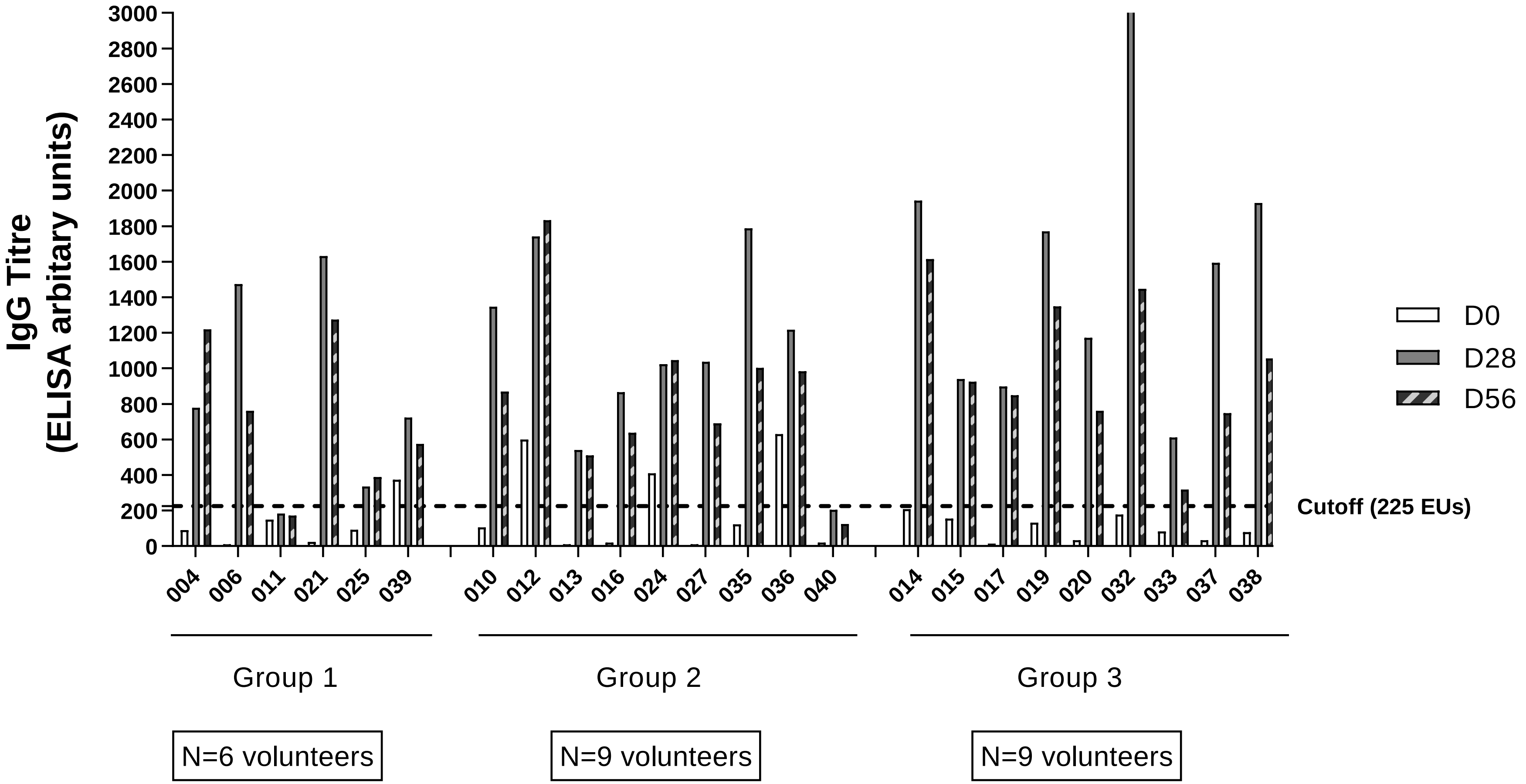

Supplementary Figure 1B: Antibody responses to ChAdOx1 MERS by dose group. Individual antibody responses for the three dose groups (Group 1, Group 2 and Group 3) at baseline as well as D28 and D56 are shown. Four volunteers (012, 024, 036 and 039) were seropositive for MERS at D0. One of these (volunteer 039) received a low dose of the vaccine (Group 1) whilst the remaining volunteers (024, 036 and 039) received a medium dose of the vaccine (Group 2).

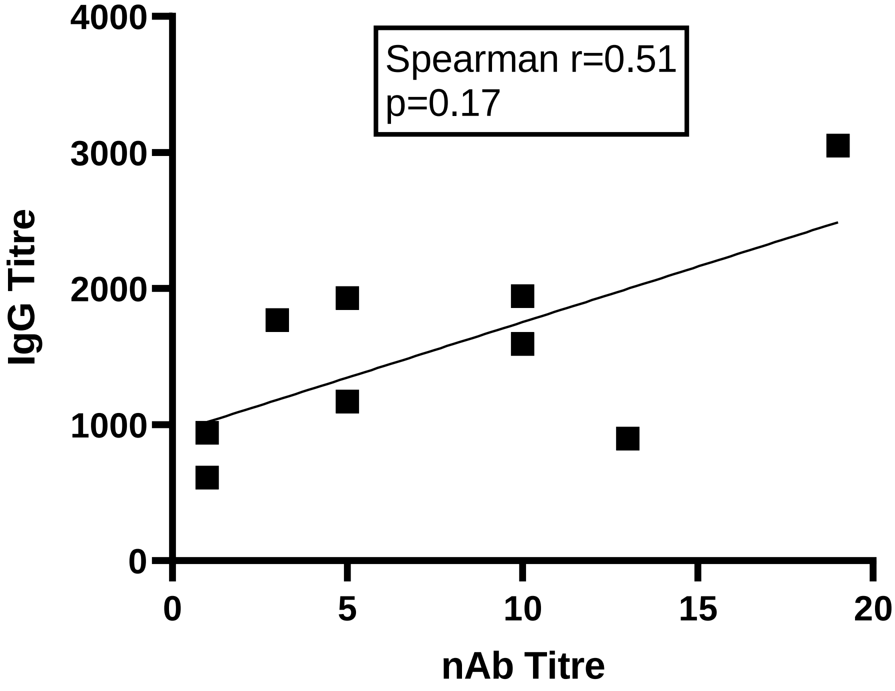

Supplementary Figure 1C: IgG versus neutralising antibody titres to ChAdOx1 MERS. Shown here is the correlation between the total IgG and neutralising antibody titres for the high dose group at the peak antibody response (D28 timepoint). Spearman r = 0.51, *p*=0.17. The line on the graph represents the linear regression line.

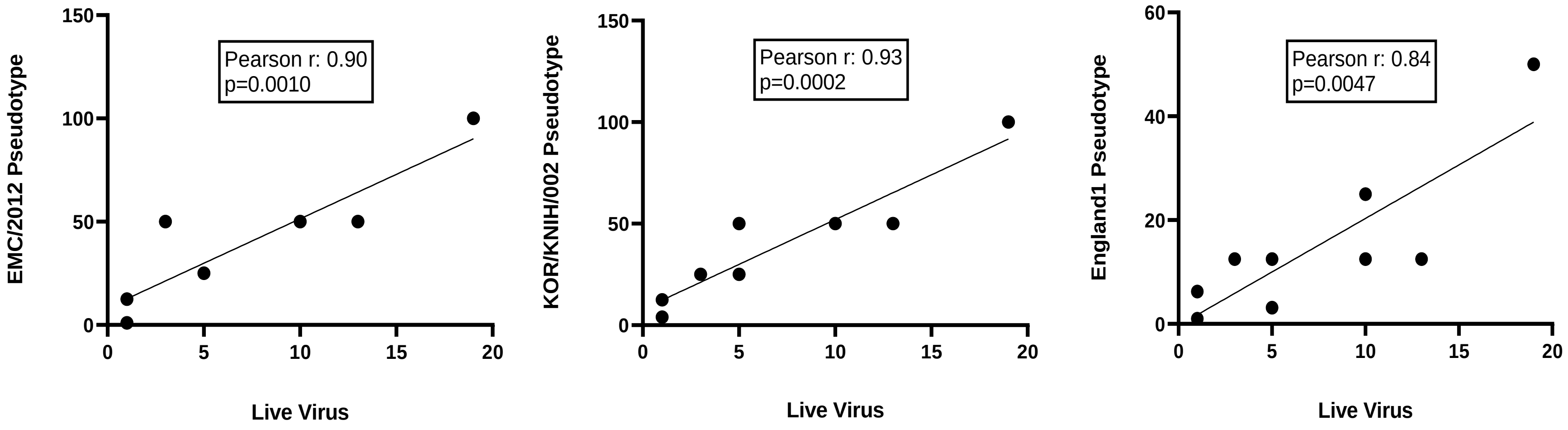

Supplementary Figure 1D: Live versus pseudotype neutralising antibody titres to ChAdOx1 MERS. Shown here is the correlation between the neutralising antibody titres for the high dose group at the peak antibody response (D28 timepoint) measured against a live MERS virus and three different pseudotyped lentiviral particles (MERS-CoV EMC/2012, KOR/KNIH/002 and England-1). The line on the graph represents the linear regression line.

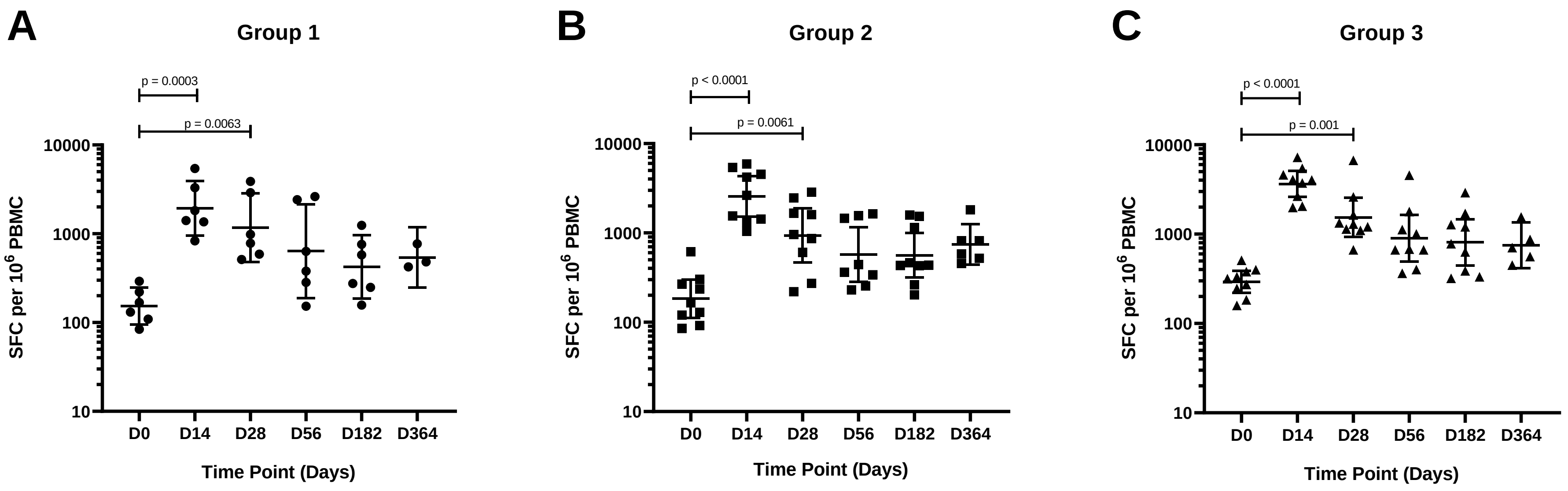

Supplementary Figure 2: T cell responses to ChAdOx1 MERS for each vaccination group. Total *ex vivo* IFN-γ ELISpot responses to MERS spike protein are shown for individual vaccinees (A) Group 1, (B) Group 2 and (C) Group 3 at all timepoints. Geometric mean with 95% CI shown. p values shown for Dunn's Multiple Comparison test.

Group 1= 5x10^9 vp, N=6 volunteers for all timepoints except D364 where N=3

Group 2 = 2.5x10^10 vp, N=9 volunteers for all timepoints except D56 (N=8) and D364 (N=6)

Group 3 = 5x10^10 vp, N=9 Volunteers for all timepoints except D364 where N=5

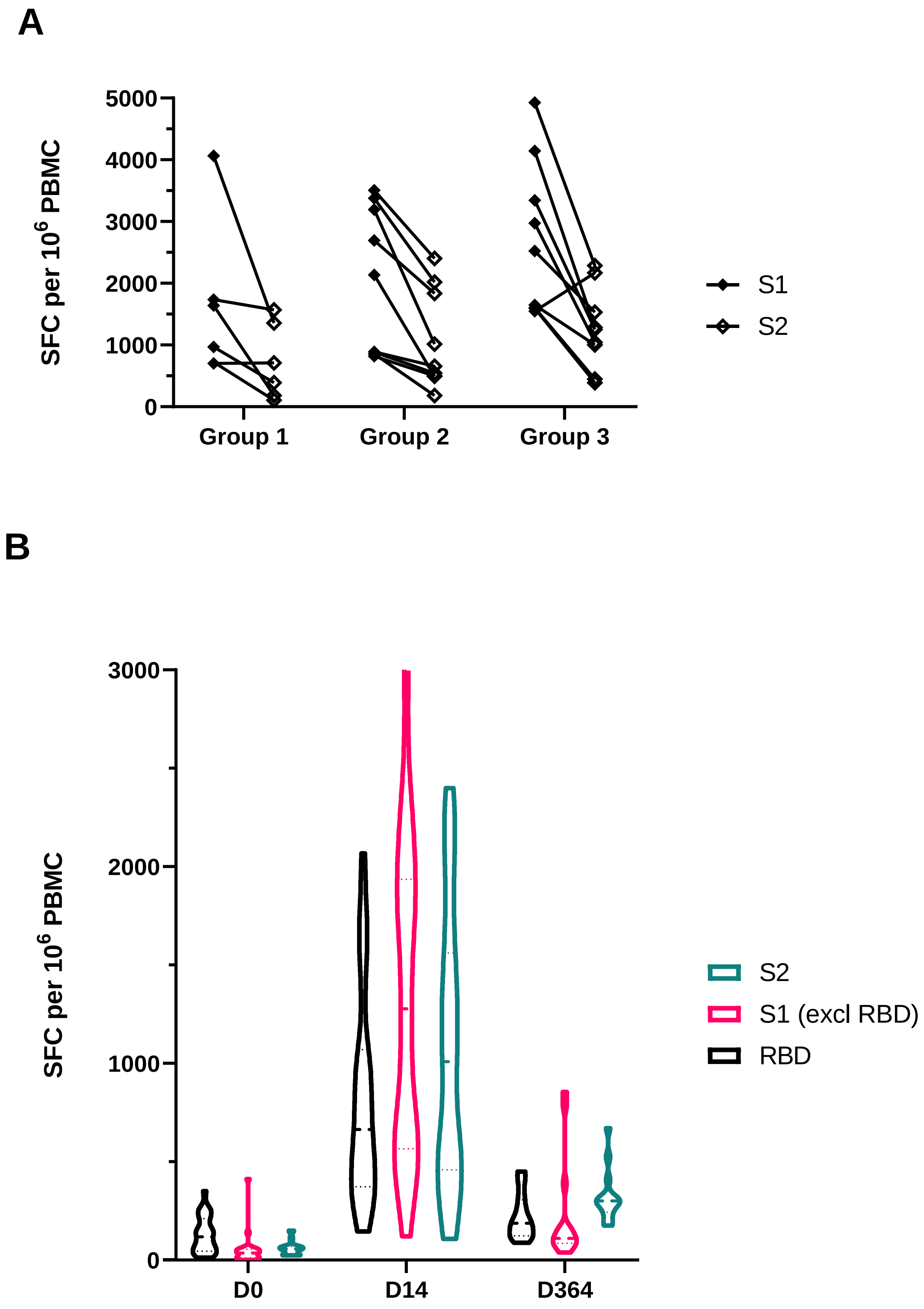

Supplementary Figure 3: T cell responses to component parts of MERS spike protein. *Ex vivo* IFN-γ ELISpot responses to peptides spanning MERS S1 and S2 (A) for individual vaccinees at D14 shown by vaccination group. S1 = pools 1 to 7 summed, S2 = pools 8 to 13 summed. (B) Violin plots for all vaccine groups combined at 3 timepoints (D0, D14 and D364). RBD = receptor binding domain, pools 4 to 6 summed, S1 (excl RBD) = pools 1-3 and 7 summed, S2 = pools 8 to 13 summed.
Group 1= 5x10^9 vp, N=6 volunteers for all timepoints except D364 where N=3

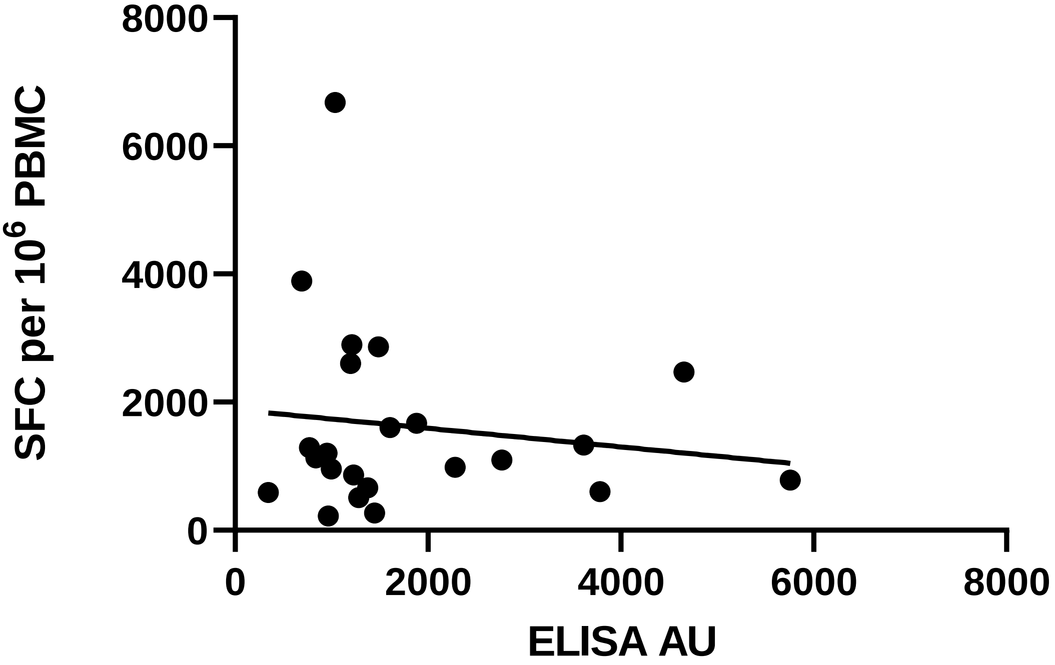

Supplementary Figure 4: **Antibody versus T cell responses to MERS spike**. Antibody responses and Total *ex vivo* IFN-γ ELISpot responses at D28 post ChAdOx1-MERS vaccination. N = 23. Line shows linear regression.

			EMC/2012	KOR/KNIH/002	England1
Group 1	MERS-06	D28	12.5	12.5	6.25
	MERS-04	D28	-	<12.5	-
	MERS-11	D28	-	<12.5	3.125
	MERS-25	D28	12.5	12.5	12.5
	MERS-21	D28	100	100	12.5
	MERS-39	D28	-	<12.5	-

			EMC/2012	KOR/KNIH/002	England1
Group 2		D0	-	-	-
	MERS-12	D28	25	25	12.5
		D56	50	50	25
	MERS-10	D28	12.5	25	6.25
	MERS-13	D28	12.5	25	6.25
	MERS-24	D28	<12.5	<12.5	<3.125
	MERS-16	D28	<12.5	<12.5	3.125
	MERS-27	D28	25	25	6.25
	MERS-35	D28	25	25	6.25
	MERS-36	D28	-	<12.5	-
	MERS-40	D28	25	25	3.125

			EMC/2012	KOR/KNIH/002	England1
Group 3	MERS-17	D28	50	50	12.5
	MERS-15	D28	12.5	12.5	6.25
	MERS-14	D28	50	50	25
	MERS-20	D28	25	25	3.125
	MERS-19	D28	50	25	12.5
	MERS-32	D28	100	100	50
	MERS-37	D28	50	50	12.5
	MERS-33	D28	-	<12.5	-
	MERS-38	D28	25	50	12.5

Supplementary Figure 5: Spike-pseudotyped neutralization titres. Sample from volunteer MER-00101012 showed higher IC50 at day56 rather than day28 unlike other individual samples. The experiments were repeated with all the day28 samples including the day56 serum of MER-00101012 individual. The pseudo-neutralizing activity (IC50) individual was confirmed to be higher at day56 (IC50=50) than that at day28 (IC50=25), which is correlated with the higher level of S1-binding antibody at day56.

UNIVERSITY OF OXFORD

A phase I study to determine the safety and immunogenicity of the candidate Middle East Respiratory Syndrome Coronavirus (MERS-CoV) vaccine ChAdOx1 MERS in UK healthy adult volunteers

Study Reference: MERS001

Protocol Number: v2.0

Date: 13th August 2018

Chief Investigator: Professor A.V.S Hill

Sponsor: University of Oxford

Funder: UK Department of Health

REC Number: 17/SC/0552

EudraCT Number: 2017-003472-31

IRAS Reference: 231515

PAGE LEFT INTENTIONALLY BLANK

**Full Study Title**	A phase I clinical trial to determine the safety and immunogenicity of the candidate MERS-CoV vaccine ChAdOx1 MERS in UK healthy adult volunteers.<br>**Study Code**: MERS001<br>**EudraCT Number**: 2017-003472-31<br>**IRAS project ID**: 231515
**Chief Investigator**	**Prof Adrian V.S. Hill**<br>Centre for Clinical Vaccinology and Tropical Medicine<br>University of Oxford,<br>Churchill Hospital, Old Road, Headington<br>Oxford, OX3 7LE<br>Email: adrian.hill@ndm.ox.ac.uk
**Trial Sites**	**Centre for Clinical Vaccinology and Tropical Medicine**<br>Churchill Hospital, Old Road, Headington<br>Oxford, OX3 7LE
**Sponsoring Institution**	**University of Oxford**<br>Clinical Trials and Research Governance<br>Joint Research Office, Block 60, Churchill Hospital,<br>Old Road, Headington, Oxford, OX3 7LE<br>Tel: 01865 572224<br>Fax: 01865 572228<br>Email: ctrg@admin.ox.ac.uk
**External Monitor**	**Clinical Trials & Research Governance**<br>Joint Research Office, Block 60,<br>Churchill Hospital,<br>Old Road, Headington, Oxford OX3 7LE
**Local Safety Monitor (Chairman of Local Safety Committee)**	**Dr Brian Angus**<br>Centre for Clinical Vaccinology and Tropical Medicine<br>Churchill Hospital, Old Road, Headington<br>Oxford, OX3 7LE<br>Tel: 01865 220289<br>Email: brian.angus@ndm.ox.ac.uk

Confidentiality Statement

This document contains confidential information that must not be disclosed to anyone other than the Sponsor, the Investigator Team, HRA, host organisation, members of the Research Ethics Committee and other regulatory bodies. This information cannot be used for any purpose other than the evaluation or conduct of the clinical investigation without the prior written consent of Professor Adrian Hill.

Statement of Compliance

The trial will be conducted in compliance with the protocol, the principles Good Clinical Practice Guideline , Medicines for Human Use (Clinical Trial) Regulations 2004 (as amended) and all other applicable regulatory requirements.

Chief Investigator Approval and Agreement

I have read the trial protocol and agree to conduct the trial in compliance with the protocol, the principles of Good Clinical Practice and all applicable regulatory requirements.

Conflict of Interest

According to the Declaration of Helsinki, 2008, I have read this protocol, and declare the following conflict of interest

Prof Adrian Hill is a scientific co-founder of, advisor to and shareholder in Vaccitech Ltd, a spin-out company that holds commercial rights to the ChAdOx1 MERS-CoV vaccine, which was in-licensed from Oxford University Innovation.

I hereby approve this version of the protocol

Adrian Hill		13th Aug 2018
**Chief Investigator**		
**Name**	**Signature**	**Date**

Modification History

Version	Date	Author(s)	Modifications
1.0	21.08.2017	Pedro Folegatti, Sarah Gilbert, Adrian Hill	N/A
1.1	16.02.2018	Pedro Folegatti	Correction of concentration from 2.06 x 10^{11} vp/mL to 1.74 x 10^{11} vp/mL of ChAdOx1 MERS
2.0	13.08.2018	Pedro Folegatti	Extended follow-up and immunology timepoints from 6 months to 1 year after enrolment.

Table of Contents

1. SYNOPSIS 24

2. ABBREVIATIONS 26

3. BACKGROUND & RATIONALE 28

3.1 Impact of MERS-CoV and the need for a vaccine 28

3.2 Progress towards a MERS vaccine 28

3.3 MERS-CoV spike protein as a vaccine antigen 29

3.4 Adenovirus-vectored Vaccines 30

3.5 ChAdOx1 31

3.6 Development of ChAdOx1 MERS 32

3.7 Preclinical Studies 32

3.7.1 Efficacy and Immunogenicity 32

3.8 Previous clinical experience 33

3.9 Rationale 35

3.10 Vaccine Development Strategy 36

4. OBJECTIVES AND ENDPOINTS 37

4.1 Primary Objective 37

4.1.1 Primary Outcome Measures 37

4.2 Secondary Objective 37

4.2.1 Secondary Outcome Measures 37

5. STUDY OVERVIEW 38

5.1 Rationale for Selected Doses 38

5.2 Study Groups 39

5.2.1 First Volunteers 39

5.2.2 Duration of study 39

5.2.3 Definition of Start and End of Trial 39

5.3 Potential Risks for volunteers 39

5.4 Known Potential Benefits 40

6. RECRUITMENT AND WITHDRAWAL OF TRIAL VOLUNTEERS 41

6.1 Volunteers 41

6.2 Informed consent 41

6.3 Inclusion and exclusion criteria 42

6.3.1 Inclusion Criteria 42

6.3.2 Exclusion Criteria 42

6.3.3 Effective contraception for female volunteers 43

6.3.4 Prevention of ‘Over Volunteering’ 44

6.3.5 Criteria for postponement of vaccination 44

6.3.6 Withdrawal of Volunteers 44

6.4 Compliance with Dosing Regime 45

6.5 Pregnancy **45**

7. CLINICAL PROCEDURES 46

7.1 Study procedures **46**

7.2 Observations **46**

7.3 Blood Tests and Urinalysis **46**

7.4 Study visits **47**

7.4.1 Screening visit 47

7.4.2 Day 0: Enrolment and Vaccination Visit 48

7.4.2.1 Vaccinations 48

7.4.2.2 Sequence of Enrolment and Vaccination of Volunteers 49

7.4.3 Subsequent visits: days 2, 7, 14, 28, 56, 182 and 364. 49

8. INVESTIGATIONAL PRODUCTS 52

8.1. Manufacturing and Presentation **52**

8.1.1 Description of ChAdOx1 MERS 52

8.1.2. ChAdOx1 MERS formulation and packaging 52

8.2 Supply **52**

8.3 Storage **52**

8.4 Administration of Investigational Medicinal Products **52**

8.5 Minimising environmental contamination with genetically modified organisms (GMO) **53**

9. ASSESSMENT OF SAFETY 54

9.1 Definitions **54**

9.1.1 Adverse Event (AE) 54

9.1.2 Adverse Reaction (AR) 54

9.1.3 Unexpected Adverse Reaction 54

9.1.4 Serious Adverse Event (SAE) 54

9.1.5 Serious Adverse Reaction (SAR) 55

9.1.6 Suspected Unexpected Serious Adverse Reaction (SUSAR) 55

9.2 Foreseeable Adverse Reactions: **55**

9.3 Expected Serious Adverse Events **55**

9.4 Causality Assessment **55**

9.5 Reporting Procedures for All Adverse Events (see SOP VC027) **56**

9.5.1 Reporting Procedures for Serious AEs (see SOP OVC005 Safety Reporting) 56

9.5.2 Reporting Procedures for SUSARS 57

9.5.3 Development Safety Update Report 57

9.6 Assessment of severity **57**

9.7 Procedures to be followed in the event of abnormal findings **58**

9.8 Local Safety Committee **58**

9.8.1 Interim Safety Reviews 59

9.9 Safety Stopping/Holding Rules **59**

9.9.1 Group holding rules ...59

10. STATISTICS ... 61

11. DATA MANAGEMENT ... 62

11.1 Data Handling ... 62

11.2 Record Keeping ... 62

11.3 Source Data and Case Report Forms (CRFs) ... 62

11.4 Data Protection ... 62

11.5 Data Quality ... 62

12. QUALITY CONTROL AND QUALITY ASSURANCE PROCEDURES ... 64

12.1 Investigator procedures ... 64

12.2 Monitoring ... 64

12.3 Protocol deviation ... 64

12.4 Audit & inspection ... 64

13. SERIOUS BREACHES ... 65

14. ETHICS AND REGULATORY CONSIDERATIONS ... 66

14.1 Declaration of Helsinki ... 66

14.2 Guidelines for Good Clinical Practice ... 66

14.3 Approvals ... 66

14.4 Volunteer Confidentiality ... 66

15. FINANCING AND INSURANCE ... 67

15.1 Financing ... 67

15.2 Insurance ... 67

15.3 Compensation ... 67

16. PUBLICATION POLICY ... 68

17. REFERENCES ... 69

1. SYNOPSIS

**Trial Title**	A phase I clinical trial to determine the safety and immunogenicity of the candidate MERS-CoV vaccine ChAdOx1 MERS in UK healthy adult volunteers.
**Trial Centre**	Centre for Clinical Vaccinology & Tropical Medicine, University of Oxford, Churchill Hospital, Old Road, Headington, Oxford, OX3 7LE
**Trial Identifier**	MERS001
**Clinical phase**	I
**Study Design**	Open–labelled, non-randomised, dose escalation, first-in-human, single centre, phase I clinical trial
**Population**	Healthy adults aged 18 – 50 years
**Planned Sample Size**	24 volunteers

Group	Dose of ChAdOx1 MERS
Group 1 (n=6)	5×10^{9} vp
Group 2 (n=9)	2.5×10^{10} vp
Group 3 (n=9)	5×10^{10} vp

**Follow-up duration**	52 weeks post vaccine administration
**Planned Trial Period**	Q4 2017 to Q4 2019
**Primary Objective**	To assess the safety profile of the candidate vaccine ChAdOx1 MERS in healthy adult volunteers
**Secondary Objective**	To assess the immunogenicity of the candidate vaccine ChAdOx1 MERS in healthy adult volunteers

**Investigational Products**	ChAdOx1 MERS, a replication-deficient simian adenoviral vector expressing the spike (S) protein of MERS Coronavirus.
**Dose per Administration**	ChAdOx1 MERS 5 x 10^9 vp<br>ChAdOx1 MERS 2.5 x 10^{10} vp<br>ChAdOx1 MERS 5 x 10^{10} vp
**Form**	Liquid (all finished products)
**Route**	Intramuscularly (IM) into the deltoid region of the arm

2. ABBREVIATIONS

AE	Adverse event
AR	Adverse reaction
CBF	Clinical Biomanufacturing Facility
CCVTM	Centre for Clinical Vaccinology and Tropical Medicine
ChAdOx1	Chimpanzee Adenovirus Ox1
ChAdOx1 MERS	Recombinant Chimpanzee Adenovirus Ox1 with MERS spike antigen
CI	Chief Investigator
CRF	Case Report Form or Clinical Research Facility
CTRG	Clinical Trials Research Governance
DSUR	Development Safety Update Report
EC	Ethics committee
ELISA	Enzyme linked immunosorbent assay
ELISpot	Enzyme linked immunospot assay
FBC	Full blood count
GCP	Good Clinical Practice
GMO	Genetically modified organism
GMP	Good Manufacturing Practice
HBsAg	Hepatitis B surface antigen
HCG	Human Chorionic Gonadotrophin
HCV	Hepatitis C virus
HIV	Human Immunodeficiency virus
HLA	Human leukocyte antigen
IB	Investigators Brochure
ICH	International Conference on Harmonisation
IM	Intramuscular
IMP	Investigational Medicinal Product
LSC	Local Safety Committee
LSM	Local Safety Monitor
MERS	Middle East Respiratory Syndrome

MERS-CoV	Middle East Respiratory Syndrome Coronavirus
MHRA	Medicines and Healthcare products Regulatory Agency
MVA	Modified Vaccinia Virus Ankara
pfu	plaque forming units
PIS	Participant information sheet
QP	Qualified Person
SAE	Serious Adverse Event
SAR	Serious Adverse Reaction
SFU	Spot forming units
SOP	Standard Operating Procedure
SUSAR	Suspected Unexpected Serious Adverse Reaction
TMF	Trial Master File
vp	Viral particles
WHO	World Health Organisation

3. BACKGROUND & RATIONALE

3.1 Impact of MERS-CoV and the need for a vaccine

The Middle East Respiratory Syndrome Coronavirus (MERS-CoV) has been identified as one of the most worrying newly emerging outbreak pathogens by the World Health Organization (WHO), the National Institute of Allergy and Infectious Diseases (NIAID), the Center for Disease Control and Prevention (CDC), Public Health England (PHE) and many other global agencies and expert groups. The disease was first described in 2012 and is now endemic in Saudi Arabia. It has since spread to different countries in the Middle East and other regions, including a recent outbreak in South Korea. More than 2000 cases of MERS-CoV with 720 deaths in 27 countries have been reported until this date (1).

The disease is caused by a Coronavirus and is spread by droplet infection of the airways. Dromedary camels are now recognised as the source of zoonotic infections and occupational exposure can lead to seroconversion. Human to human transmission, specially in hospital environments, have been responsible for the majority of cases seen in recent outbreaks. The clinical spectrum of MERS-CoV infection varies from asymptomatic or mild respiratory symptoms to severe acute respiratory disease and death. Common clinical symptoms include fever, cough and shortness of breath. Pneumonia is a common finding, but it might not always be present. Gastrointestinal symptoms, including diarrhoea, have also been reported. Severe illness can cause respiratory failure that requires mechanical ventilation and support in an intensive care unit. MERS-CoV has a reported case fatality rate of approximately 35%. The virus appears to cause more severe disease in the elderly, immunosuppressed and those with chronic diseases such as cardiovascular disease and diabetes (2-5).

The disease has been chosen as a very high priority disease for accelerated vaccine development by the WHO, international vaccine experts and by members of the UK Vaccines Research and Development network (6). Vaccines are required for both camels and people. Vaccinating camels in the Middle East, combined with strict controls on import of camels from other countries associated with effective hospital infection prevention and control measures could eventually lead to the eradication of the disease in the Middle East, but it will take time to achieve this. In addition the vaccination of workers who are occupationally exposed to camels would prevent them from becoming infected and limit the transmission of the virus to the wider population, in particular those at an increased risk of death such as the elderly or immunocompromised.

The dipeptidyl peptidase 4 (DPP4) receptor is used by the MERS-CoV virus during infection and is highly conserved between Camels and Humans. The MERS-CoV spike (S) protein is a characteristic structural component of the virion membrane and its S1 domain mediates binding to DDP4. The spike protein has been chosen as the target antigen for use in the replication-deficient simian adenovirus developed by the University of Oxford, ChAdOx1 vaccine vector. ChAdOx1 has shown successful results in the development of Oxford lead vaccines which have gone onto enter phase I trials within the UK (7). In this study we propose the manufacture to GMP of the ChAdOX1 MERS vaccine and its use in a small open labelled phase I trial in Oxford.

3.2 Progress towards a MERS vaccine

Global efforts to develop a coronavirus vaccine faded in the aftermath of Severe Acute Respiratory Syndrome Coronavirus (SARS-CoV) pandemic but has since gained renewed momentum in the face of the current MERS-CoV outbreak. Most of the developed vaccines were based on the S surface

glycoprotein, the primary target for neutralizing antibodies during any natural coronavirus infection. A number of preclinical and clinical studies showed that the SARS-CoV S1 protein subunit, and specifically the RBD at its core, could serve as a dominant target for neutralizing antibodies in mice, non-human primates, and humans. S1, therefore, became the basis for a number of promising SARS-CoV vaccine candidates (8).

The S1 protein subunit and the Receptor Binding Domain (RBD) have also been the basis for several MERS-CoV vaccine candidates. Both constructs have elicited neutralizing antibodies of high potency across multiple viral strains. Despite their demonstrated immunogenicity in animal models and anticipated safety in humans, RBD or S1-subunit based vaccine candidates are limited in their epitope breadth. Vaccine candidates that elicit a more diverse antibody repertoire as well as a robust cellular immuneresponse may offer the advantage of broader and more durable protection (8).

Live attenuated viruses have historically been among the most immunogenic platforms available, as they have the capacity to present multiple antigens across the viral life cycle in their native conformations. However, manufacturing live-attenuated viruses requires complex containment and biosafety measures. Furthermore, live-attenuated viruses carry the risks of inadequate attenuation causing disseminated disease, particularly in immunocompromised hosts. Given that moderately immuno-compromised adults with co-morbidities have suffered the most severe MERS-CoV disease, making a live-attenuated virus vaccine is a less viable option. Replication competent viral vectors could pose a similar threat for disseminated disease in the immuno-suppressed. Replication deficient vectors, however, avoid that risk while maintaining the advantages of native antigen presentation, elicitation of T cell immunity and the ability to express multiple antigens (8).

Several recombinant DNA, protein and viral vectored MERS-CoV candidate vaccines have been developed and tested in animal models (mice, non-human primates and camels) with varied efficacy results. Recently, a recombinant MVA encoding the full length Spike protein antigen (S) showed partial efficacy by significantly reducing MERS-CoV viral shedding in a camel challenge study. (9, 10)

The first MERS-CoV vaccine to be used in humans has recently entered a phase I dose ranging safety study in January 2016. The GLS-5300, a DNA plasmid vaccine that expresses the MERS-CoV spike (S) glycoprotein, is being administered to 75 healthy adult volunteers in the USA, by the Walter Reed Army Institute of Research. Safety and immunogenicity data are expected to be reported by the end of 2017.

3.3 MERS-CoV spike protein as a vaccine antigen

Coronaviruses (CoVs) are spherical and enveloped viruses with large, unsegmented, single positive RNA genomes. One-third of the genome is responsible for coding the structural proteins: spike (S) glycoprotein, small envelope protein (E), integral membrane protein (M), and genome-associated nucleocapsid protein (N). The proteins E, M, and N are mainly responsible for the assembly of the virions, while the S protein is involved in receptor binding and bears membrane fusion capabilities during CoVs infection. Thus, the S protein has an essential role in virus entry and determines tissue and cell tropism, as well as host range (11).

S is a type I, trimeric, transmembrane protein located at the surface of the viral envelope, giving rise to spikeshaped protrusions from the virion. S is 1353 amino acids in length, heavily glycosylated (with 21 predicted N-linked glycosylation sites), and consists of a large ectodomain and a short

cytosolic tail. The S proteins of CoVs can be divided into two functional subunits: the N-terminal S1 subunit forms the globular head, and the membrane-embedded C-terminal S2 (11). S1 and S2 subunits are respectively, responsible for cellular receptor DPP4 binding via the RBD, and fusion of virus and cell membranes, thereby mediating the entry of MERS-CoV into the target cells. The MERS-CoV RBD consists of a core structure, which is homologous to that of the SARS-CoV S protein RBD, and a receptor-binding motif, which is unique to MERS-CoV, thus determining viral pathogenesis and receptor recognition (12).

The roles of S in receptor binding and membrane fusion make it a perfect target for vaccine and antiviral development. Previous studies on SARS-CoV reveal that vaccines based on the S protein can induce antibodies to block virus binding and fusion or neutralize virus infection (11). ChAdOx1 MERS expresses a codon-optimised coding sequence for the Spike protein from the MERS-CoV isolate Camel/Qatar_2_2014 (GenBank:KJ650098.1).

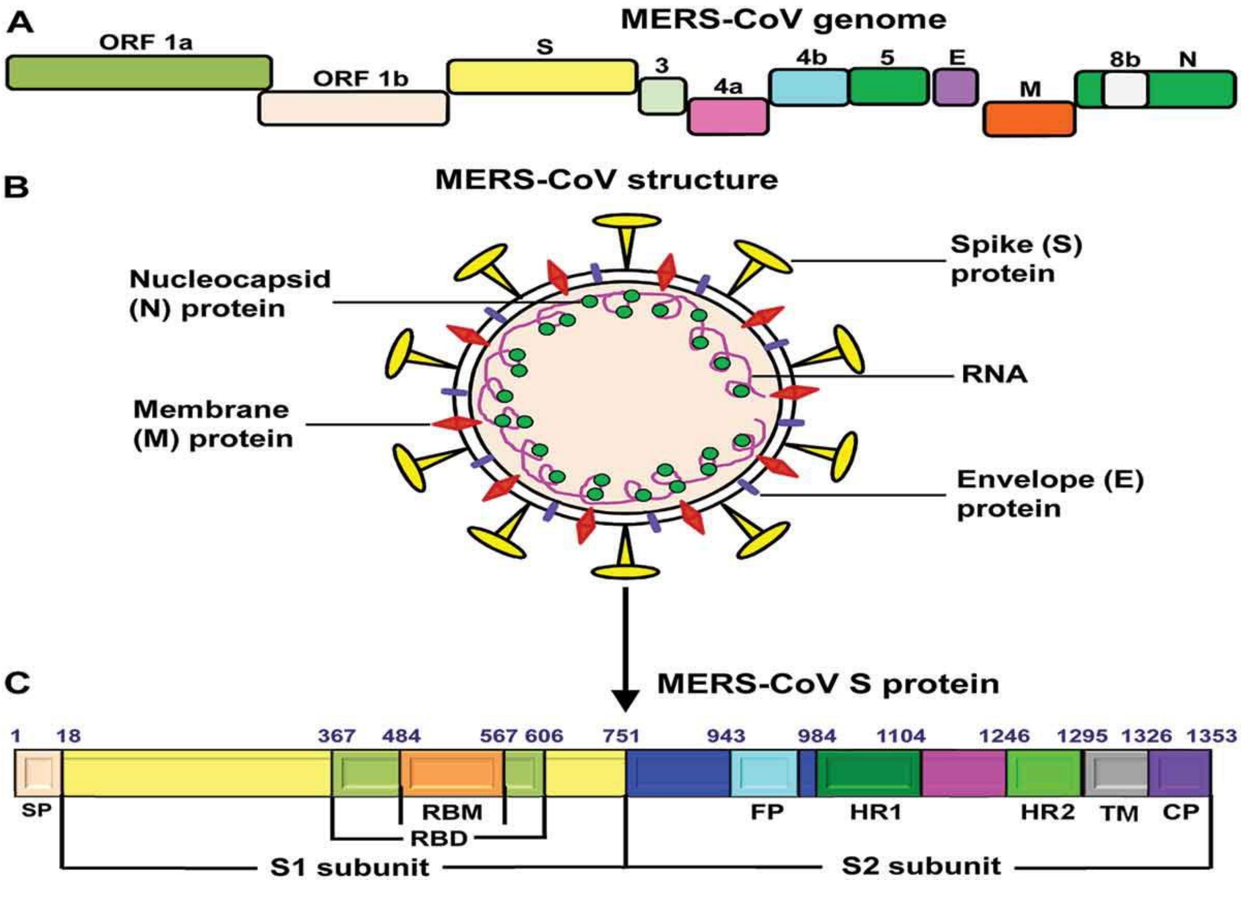

Figure 1. MERS-CoV structure. Published in: Lanying Du; Wanbo Tai; Yusen Zhou; Shibo Jiang; Expert Review of Vaccines (12)

3.4 Adenovirus-vectored Vaccines

Adenoviruses are attractive vectors for human vaccination. They possess a stable genome so that inserts of foreign genes are not deleted and they can infect large numbers of cells without any evidence of insertional mutagenesis.

Replication defective adenovirus can be engineered by deletion of genes from the E1 locus, which is required for viral replication, and these viruses can be propagated easily with good yields in cell lines expressing E1 from AdHu5 such as human embryonic kidney cells 293 (HEK 293 cells) (13).

Previous mass vaccination campaigns in over 2 million adult US military personnel using orally administered live human adenovirus serotype 4 and 7 have shown good safety and efficacy data (14). Human adenoviruses are under development as vectors for malaria, HIV and hepatitis C vaccines, amongst others. They have been used extensively in human trials with excellent safety profile mainly as vectors for HIV vaccines.

A limiting factor to widespread use of human adenovirus as vaccine vectors has been the level of anti-vector immunity present in humans where adenovirus is a ubiquitous infection. The prevalence of immunity to human adenoviruses prompted the consideration of simian adenoviruses as vectors, as they exhibit hexon structures homologous to human adenoviruses (15). Simian adenoviruses are not known to cause pathological illness in humans and the prevalence of antibodies to chimpanzee origin adenoviruses is less than 5% in humans residing in the US.

In chimpanzee adenoviruses, the E1 locus can be deleted to render viruses replication deficient and allow transcomplementation on an E1 AdHu5 complementing cell line (16). Whilst they exhibit hexon structures homologous to that of human adenoviruses (17), the lack of sequence homology at the E1 flanking sequence prevents homologous recombination and production of replication competent virus (18)

Chimpanzee adenoviral vectors can be manufactured cost-effectively and are now in clinical development as possible vaccines against malaria, HIV, tuberculosis, influenza, hepatitis C, RSV, Cancer and Ebola.

3.5 ChAdOx1

ChAdOx1 is a novel recombinant chimpanzee adenovirus designed as a vaccine vector, developed by The Jenner Institute at the University of Oxford. This viral vector has been used by researchers at the University of Oxford to produce a number of vaccines expressing a range of different antigens. Three phase I clinical trials have been completed in the UK using ChAdOx1 with different inserts (two influenza trials and one TB trial).

ChAdOx1 is produced from a replication-deficient (E1 and E3 deleted) simian adenovirus and it has been described by Dicks et al (19). The vector was constructed in a bacterial artificial chromosome (BAC) to facilitate genetic manipulation of genomic clones with improved stability and flexibility. Cellular immunogenicity of recombinant E1 E3-deleted ChAdOx1 was comparable to that of other species E derived chimpanzee adenovirus vectors including ChAd63, the first simian adenovirus vector to enter clinical trials in humans. The E1 region is essential for viral replication, hence the ability to delete E1 renders the new vector immediately replication incompetent. The deletion of the non-essential adenovirus E3 region increases the insert capacity of the new vector by approximately 5kb. It is known that the proteins encoded by the E4 region of adenoviruses interact with E1 during viral replication, and the imperfect interaction between the gene products of the AdHu5 E1 gene produced by HEK293 cells and simian E4 gene products has been found to result in impaired viral replication in this cell line, and consequently lower virus yields. In ChAdOx1, Ad5 E4Orf4 has been inserted to replace the homologous simian virus coding sequence, resulting in improved viral replication during vaccine production. Since no replication of the virus takes place after

immunization, this replacement has no effect on immunogenicity of the viral vector. Insertion of recombinant antigens at the E1 locus is performed using Gateway® site specific recombination technology (Invitrogen).

3.6 Development of ChAdOx1 MERS

ChAdOx1 MERS encodes the Spike (S) surface glycoprotein of the coronavirus. A genomic clone of ChAdOx1 MERS was prepared by Gateway® recombination between an entry plasmid containing the codon-optimised coding sequence for Spike protein from the MERS-CoV isolate Camel/Qatar_2_2014 (GenBank:KJ650098.1), and the E1-and E3-deleted ChAdOx1 destination vector.

3.7 Preclinical Studies

3.7.1 Efficacy and Immunogenicity

Mice (balb/c) were immunised with ChAdOx1 or MVA vectored vaccines expressing MERS-CoV Spike protein. Serum samples were taken after 28 days and endpoint titres measured by ELISA. This study showed that a single dose of ChAdOx1 results in equivalent immunogenicity to two doses of MVA (20).

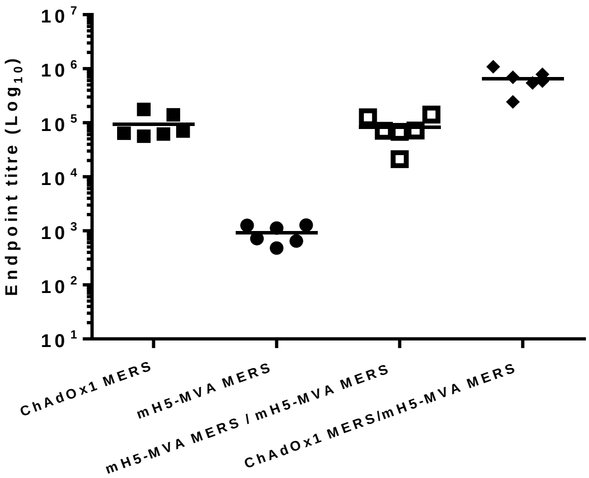

Figure 2. Immunogenicity of viral vectored vaccines MERS vaccines in mice.

An efficacy preclinical study has been conducted where mice transgenic for the hDPP4 receptor were immunised with a single dose of ChAdOx1 MERS by either intranasal or intramuscular injection. The control ChAdOx1 vaccine expressed eGFP as the vaccine antigen. Serum neutralising titres were measured 28 days after vaccination, when the mice were then challenged with MERS CoV by intranasal inoculation. The results showed that mice immunised with the MERS vaccine by either route were completely protected against MERS-CoV infection. No virus was detected in the lungs of the mice receiving the MERS vaccine and they all survived, whereas all of the sham-vaccinated mice succumbed to infection within 8 days (Vincent Munster,unpublished data).

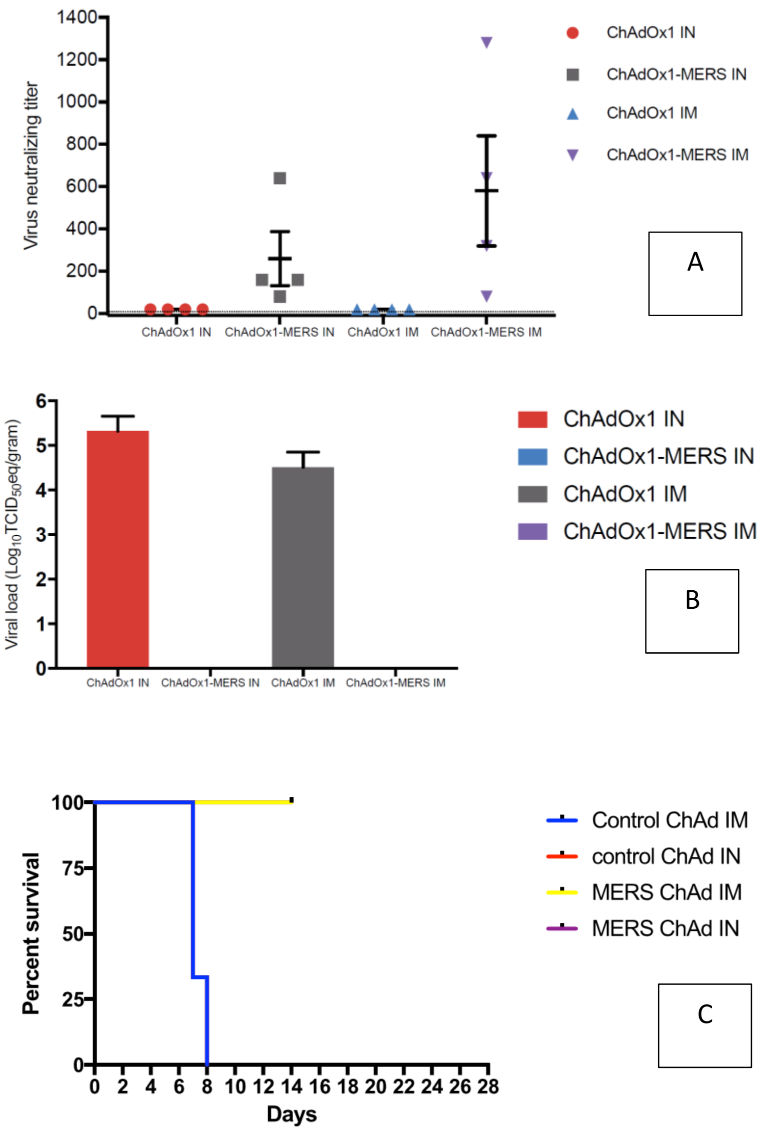

Figure 3. A. Virus neutralising titres in mice amongst ChAdOx1 MERS and controls administered via intranasal or intramuscularly. **B**. Viral load after MERS-CoV challenge. **C** Survival amongst ChAdOx1 MERS and control mice after intranasal MERS-CoV challenge.

3.8 Previous clinical experience

This will be the first-in-human study employing ChAdOx1 MERS. However, ChAdOx1 vectored vaccines expressing different inserts have previously been used in 161 healthy volunteers taking part in clinical trials conducted by the University of Oxford in the UK (table 1).

ChAdOx1 encoding the influenza fusion protein NP+M1 has been safely administered to 84 healthy adult volunteers in the UK in two completed clinical trials conducted at The Jenner Institute (FLU004 and FLU005). FLU004 was a phase I, open-label, non-randomised dose escalation study of ChAdOx1 NP+M1. The vaccine was safe, well tolerated and immunogenic, inducing ELISpot responses at all doses. The dose of 2.5×10^{10} vp was chosen for further studies of ChAdOx1 NP+M1 (7).

FLU005 was a multicentre phase I, randomised study to determine the safety and immunogenicity of vaccination regimens employing the candidate influenza vaccines MVA-NP+M1 and ChAdOx1 NP+M1. Sixty-nine healthy adult volunteers have received ChAdOx1 NP+M1 at a dose of 2.5 x 10^{10} vp. Administrations of ChAdOx1 NP+M1 and MVA-NP+M1 vaccines were found to be safe and well-tolerated, in agreement with our previous studies (7, 21-23). The majority of adverse events were mild to moderate in nature and lasted for 1-2 days. The most common local adverse event was arm pain at the site of injection and the most common systemic adverse event was mild fatigue and headache.

TB034 was an open-label, phase I clinical trial in which 42 healthy adult volunteers received the ChAdOx1 viral vector expressing the *Mycobacterium tuberculosis* antigen 85A (ChAdOx1 85A). No major safety concerns associated with ChAdOx1 85A administration have been reported.

ChAdOx1 5T4 has been given in the VANCE01 study which is an ongoing first-in-man open label randomized phase I study to determine the safety and immunogenicity of heterologous prime boost ChAd-MVA vaccination against oncofetal antigen 5T4. To date, 34 participants have received the ChAdOx1 5T4 vaccine at a dose of 2.5 x 10^{10} vp and only mild AEs related to the vaccination have been reported

VAC067 is an ongoing first-in-man study of the ChAdOx1 viral vector expressing dual second generation liver-stage malaria antigens LSA1 and LSAP2 (ChAdOx1 LS2). No significant safety concerns have been reported until this date.

None of the above mentioned clinical trials reported serious adverse events associated with the administration of ChAdOx1, which was shown to have a good safety profile.

Table 1. Clinical experience with ChAdOx1 viral vector vaccines.

Country	Trial	Vaccine	Age	Route	Dose	Number of Volunteers (Received ChAdOx1)
UK	FLU004	ChAdOx1 NP+M1	18-50	IM	5x10^{8} vp	3
					5x10^{9} vp	3
					2.5x10^{10} vp	3
					5x10^{10} vp	6
UK	FLU005	ChAdOx1 NP+M1<br>MVA NP+M1 (week 8)	18-50	IM	2.5x10^{10} vp	12
		ChAdOx1 NP+M1<br>MVA NP+M1 (week 52)	18-50	IM	2.5x10^{10} vp	12
		MVA NP+M1<br>ChAdOx1 NP+M1 (week 8)	18-50	IM	2.5x10^{10} vp	12
		MVA NP+M1<br>ChAdOx1 NP+M1 (week 52)	18-50	IM	2.5x10^{10} vp	9
		ChAdOx1 NP+M1	>50	IM	2.5x10^{10} vp	12
		ChAdOx1 NP+M1<br>MVA NP+M1 (week 8)	>50	IM	2.5x10^{10} vp	12
UK	TB034	ChAdOx1 85A	18-50	IM	5x10^{9} vp	6
					2.5x10^{10} vp	12
		ChAdOx1 85A<br>MVA85A (week 8)	18-50	IM	2.5x10^{10} vp	12
UK	VANCE01<br>*(ongoing)*	ChAdOx1.5T4<br>MVA.5T4	18 – 75	IM	2.5x10^{10} vp	34 (as of Sep 2017)
UK	VAC067	ChAdOx1 LS2	18-45	IM	5x10^{9} vp	3
					2.5x10^{10} vp	10

3.9 Rationale

MERS-CoV is an emerging zoonotic viral disease considered a global threat and listed as a priority pathogen for urgent Research and Development. The recent MERS-CoV outbreaks in the Middle East (from 2012 and still ongoing) and South Korea (2015) have caused a total of 720 deaths representing a case fatality rate of approximately 35% and imported cases have now been reported in 27 countries.

Chimpanzee adenovirus vaccine vectors have been safely administered to thousands of people using a wide range of infectious disease targets including malaria (24), HIV (25), tuberculosis, influenza (7), hepatitis C (26), RSV (27) and most recently Ebola (28). ChAdOx1 viral vectored vaccines have shown to be both safe and immunogenic in previous clinical trials in the UK (FLU004, FLU005, TB034, VANCE001 and VAC067). Single-dose immunisation with ChAdOx1 MERS vaccine has shown to elicit high levels of neutralising antibody in animal models.

Finally, the One Health vaccine development approach used here, by which the same vaccine is co-developed for humans and susceptible animal species, is well suited to many emerging outbreak pathogens, most of which involve zoonotic transmission (29). The approach allows the possibility of cost reductions for the final product by increasing the scale of manufacture (30). Ultimately the vaccine could be licensed for use in camels in the Middle East and North Africa. If licensed, human

vaccines could be deployed for occupationally exposed individuals such as camel workers and health care professionals, with stockpiles available for use in the case of an outbreak.

3.10 Vaccine Development Strategy

The data from this first in human study will then be used to support a further phase I and phase II study with our collaborators based in the Middle East. This study would be run in parallel with a study in camels with our collaborators at Qassim University, Saudi Arabia. Testing of the MERS vaccine in camels is funded by the Liverpool School of Tropical Medicine Confidence in Concept Scheme.

4. OBJECTIVES AND ENDPOINTS

The number of volunteers has been chosen to generate adequate safety and immunogenicity data to meet these objectives, whilst minimising the number of volunteers exposed to a new vaccination regimen.

4.1 Primary Objective

To assess the safety and tolerability of ChAdOx1 MERS in healthy volunteers.

4.1.1 Primary Outcome Measures

The specific endpoints for safety and reactogenicity will be actively and passively collected data on adverse events.

The following parameters will be assessed for all study groups

- Occurrence of solicited local reactogenicity signs and symptoms for 7 days following the vaccination
- Occurrence of solicited systemic reactogenicity signs and symptoms for 7 days following the vaccination
- Occurrence of unsolicited adverse events for 28 days following the vaccination
- Change from baseline for safety laboratory measures
- Occurrence of serious adverse events during the whole study duration

Volunteers will undergo clinical follow up for adverse events for a further 364 days following completion of the vaccination regimen. SAEs will be collected throughout the study. The duration of follow up reflects the desire to obtain longer term safety data with the first use of ChAdOx1 MERS in humans.

4.2 Secondary Objective

To assess the cellular and humoral immunogenicity of ChAdOx1 MERS in healthy adult volunteers.

4.2.1 Secondary Outcome Measures

Measures of immunogenicity to the ChAdOx1 MERS vaccine may include:

- ELISA to quantify antibodies to MERS Spike protein antigen
- Ex vivo ELISpot responses to MERS Spike protein antigen

Other exploratory immunology may be carried out in collaboration with other specialist laboratories, including laboratories outside of Europe. This would involve transfer of serum/plasma and/or peripheral blood mononuclear cells (PBMC), but samples would be anonymised. Volunteers will be consented for this.

5. STUDY OVERVIEW

This is a first-in-human, open-label, dose escalation, phase I clinical trial to assess the safety and immunogenicity of the candidate ChAdOx1 MERS vaccine in healthy volunteers aged 18-50. The vaccine will be administered intramuscularly.

Volunteers will be recruited and vaccinated at the CCVTM, Oxford. There will be 3 study groups and a total of 24 volunteers will be enrolled (table 2). Staggered enrolment will apply for the first three volunteers within each group. Volunteers will be first recruited into Group 1 and subsequently into Groups 2 and 3 following interim clinical safety reviews (see section 7.4.2). Volunteers will be allocated to a study group by selecting eligible volunteers for enrolment following screening.

5.1 Rationale for Selected Doses

Doses to be administered in this trial have been selected on the basis of clinical experience with the ChAdOx1 adenovirus vector expressing different inserts and similar adenovirus vectored vaccines (eg. ChAd63).

A first-in-man dose escalation study using the ChAdOx1 vector encoding an influenza antigen (FLU004), safely administered ChAdOx1 NP+M1 at doses ranging from 5×10^{8} to 5×10^{10} vp. Subsequent review of the data identified an optimal dose of 2.5×10^{10} vp balancing immunogenicity and reactogenicity. This dose has subsequently been given to over 100 volunteers in numerous larger phase 1 studies at the Jenner Institute (FLU005, TB034 VANCE01 and VAC067) and ChAdOx1 vectored vaccines have thus far demonstrated to be very well tolerated. The vast majority of AEs have been mild-moderate and there have been no SARs until this date.

Another simian adenovirus vector (ChAd63) has been safely administered at doses up to 2×10^{11} vp with an optimal dose of 5×10^{10} vp, balancing immunogenicity and reactogenicity.

As this is a first-in-human assessment of the MERS-CoV S antigenic insert, the first dose of ChAdOx1 MERS proposed in this study (5×10^{9} vp) is therefore at least 10 fold less than what this new insert is expected to be tolerated (5×10^{10} vp). Doses will be gradually increased aiming to provide an optimal dose of ChAdOx1 MERS considering the tolerability, reactogenicity and immunogenicity profiles.

5.2 Study Groups

Table 2. Study Groups

Group	Single Dose ChAdOx1 MERS	Route
Group 1 (n=6)	5×10^{9} vp	IM
Group 2 (n=9)	2.5×10^{10} vp	IM
Group 3 (n=9)	5×10^{10} vp	IM

5.2.1 First Volunteers

Volunteers will be enrolled and doses will be escalated according to the plan outlined below.

The first volunteer in the study will receive 5×10^{9} vp of ChAdOx1 MERS (group 1). This volunteer will be vaccinated ahead of any other volunteers and the profile of adverse events will be examined after 48h (±24h). Provided there are no safety concerns as assessed by the Chief Investigator (CI) and the Chairman of Local Safety Committee (LSC), another 2 volunteers will be vaccinated at the same dose after at least 48 hours has elapsed following vaccination of the first volunteer and at least 1 hour apart from each other. An independent safety review will be conducted by the Chairman of LSC after vaccination of the first 3 volunteers in group 1. This review will include the results of safety blood tests at day 7 post vaccination and an assessment of the profile of the adverse events reported. The CI and the Chairman of LSC will be asked to provide the decision on whether to proceed with vaccinations of the remaining participants in group 1 (for immunology data) and the first volunteer to receive the next incremental dose in group 2. If there are no safety concerns, the remaining volunteers in Group 1 and the first volunteer in group 2 may be vaccinated.

The same procedure will apply for each of the first 3 volunteers enrolled at higher dosage groups and prior to dose escalation (groups 2 and 3).

5.2.2 Duration of study

The total duration of the study will be 52 weeks from the day of enrolment for all volunteers.

5.2.3 Definition of Start and End of Trial

The start of the trial is defined as the date of the first vaccination of the first volunteer. The end of the trial is the date of the last visit of the last volunteer.

5.3 Potential Risks for volunteers

The potential risk to participants is considered as low. The potential risks are those associated with phlebotomy and vaccination. In general, recombinant adenoviral vectors are safe. Similar vaccines encoding different antigens have been given to several thousand volunteers (including children) with a good safety profile.

<u>Phlebotomy:</u>

The maximum volume of blood drawn over the study period (approximately 335mL) should not compromise these otherwise healthy volunteers. There may be minor bruising, local tenderness or pre-syncopal symptoms associated with venepuncture, which will not be documented as AEs if they occur.

Vaccination:

ChAdOx1 MERS has not been used in humans before and therefore will be initially administered at the lower dose of 5×10^9 vp before progressing to the higher doses of 2.5×10^{10} and 5×10^{10} in Groups 2 and 3. Potential expected risks from vaccination include local effects such as pain, redness, warmth, swelling, tenderness or itching. Systemic reactions that could potentially occur following immunisation with a recombinant adenovirus vaccine include a flu-like illness with feverishness, fatigue, malaise, arthralgia, myalgia and headache.

As with any vaccine, Guillain-Barré syndrome or immune-mediated reactions that can lead to organ damage may occur, but this should be extremely rare. Serious allergic reactions including anaphylaxis could also occur and for this reason volunteers will be vaccinated in a clinical area where Advanced Life Support trained physicians, equipment and drugs are immediately available for the management of any serious adverse reactions (SAR).

5.4 Known Potential Benefits

Volunteers will not benefit directly from participation in this study. However, it is hoped that the information gained from this study will contribute to the development of a safe and effective MERS-CoV vaccine regime. The only benefits for participants would be information about their general health status.

6. RECRUITMENT AND WITHDRAWAL OF TRIAL VOLUNTEERS

6.1 Volunteers

Volunteers may be recruited by use of an advertisement +/- registration form formally approved by the ethics committee(s) and distributed or posted in the following places:

- In public places, including buses and trains, with the agreement of the owner/proprietor.
- In newspapers or other literature for circulation.
- On radio via announcements.
- On a website or social media site operated by our group or with the agreement of the owner or operator (including on-line recruitment through our web-site).
- By e-mail distribution to a group or list only with the express agreement of the network administrator or with equivalent authorisation.
- By email distribution to individuals who have already expressed an interest in taking part in any clinical trial at the Oxford Vaccine Centre.
- On stalls or stands at exhibitions or fairs.
- Via presentations (e.g. presentations at lectures or invited seminars).
- Direct mail-out: This will involve obtaining names and addresses of adults via the most recent Electoral Roll. The contact details of individuals who have indicated that they do not wish to receive postal mail-shots would be removed prior to the investigators being given this information. The company providing this service is registered under the Data Protection Act 1998. Investigators would not be given dates of birth or ages of individuals but the list supplied would only contain names of those aged between 18-50 years (as per the inclusion criteria).
- Oxford Vaccine Centre databases: We may contact individuals from databases of groups within the CCVTM (including the Oxford Vaccine Centre database) of previous trial participants who have expressed an interest in receiving information about all future studies for which they may be eligible.

6.2 Informed consent

All volunteers will sign and date the informed consent form before any study specific procedures are performed. The information sheet will be made available to the volunteer at least 24 hours prior to the screening visit. At the screening visit, the volunteer will be fully informed of all aspects of the trial, the potential risks and their obligations. The following general principles will be emphasised:

- Participation in the study is entirely voluntary
- Refusal to participate involves no penalty or loss of medical benefits
- The volunteer may withdraw from the study at any time
- The volunteer is free to ask questions at any time to allow him or her to understand the purpose of the study and the procedures involved
- The study involves research of an investigational vaccine

- There is no direct benefit from participating
- The volunteer's GP will be contacted to corroborate their medical history
- The volunteer's blood samples taken as part of the study will be stored indefinitely and samples may be sent outside of the UK and Europe to laboratories in collaboration with the University of Oxford. These will be anonymised.

The aims of the study and all tests to be carried out will be explained. The volunteer will be given the opportunity to ask about details of the trial, and will then have time to consider whether or not to participate. If they do decide to participate, they will sign and date two copies of the consent form, one for them to take away and keep, and one to be stored in the case report form (CRF) – this is a paper or electronic document used to collect data relating to a particular volunteer. These forms will also be signed and dated by the Investigator.

6.3 Inclusion and exclusion criteria

This study will be conducted in healthy adults, who meet the following inclusion and exclusion criteria:

6.3.1 Inclusion Criteria

The volunteer must satisfy all the following criteria to be eligible for the study:

1. Healthy adults aged 18 to 50 years
2. Able and willing (in the Investigator's opinion) to comply with all study requirements
3. Willing to allow the investigators to discuss the volunteer's medical history with their General Practitioner
4. For females only, willingness to practice continuous effective contraception (see below) during the study and a negative pregnancy test on the day(s) of screening and vaccination
5. Agreement to refrain from blood donation during the course of the study
6. Provide written informed consent

6.3.2 Exclusion Criteria

The volunteer may not enter the study if any of the following apply:

1. Participation in another research study involving receipt of an investigational product in the 30 days preceding enrolment, or planned use during the study period
2. Prior receipt of an investigational vaccine likely to impact on interpretation of the trial data (e.g. Adenovirus vectored vaccine).
3. Administration of immunoglobulins and/or any blood products within the three months preceding the planned administration of the vaccine candidate
4. Any confirmed or suspected immunosuppressive or immunodeficient state, including HIV infection; asplenia; recurrent, severe infections and chronic (more than 14 days)

immunosuppressant medication within the past 6 months (inhaled and topical steroids are allowed)

5. History of allergic disease or reactions likely to be exacerbated by any component of the vaccine
6. Any history of hereditary angioedema, acquired angioedema, or idiopathic angioedema.
7. Any history of anaphylaxis in relation to vaccination
8. Pregnancy, lactation or willingness/intention to become pregnant during the study
9. History of cancer (except basal cell carcinoma of the skin and cervical carcinoma in situ)
10. History of serious psychiatric condition likely to affect participation in the study
11. Bleeding disorder (eg. factor deficiency, coagulopathy or platelet disorder), or prior history of significant bleeding or bruising following IM injections or venepuncture
12. Any other serious chronic illness requiring hospital specialist supervision
13. Suspected or known current alcohol abuse as defined by an alcohol intake of greater than 42 units every week
14. Suspected or known injecting drug abuse in the 5 years preceding enrolment
15. Seropositive for hepatitis B surface antigen (HBsAg)
16. Seropositive for hepatitis C virus (antibodies to HCV)
17. Any clinically significant abnormal finding on screening biochemistry or haematology blood tests or urinalysis
18. Any other significant disease, disorder or finding which may significantly increase the risk to the volunteer because of participation in the study, affect the ability of the volunteer to participate in the study or impair interpretation of the study data
19. Inability of the study team to contact the volunteer's GP to confirm medical history and safety to participate
20. Prior exposure to MERS-CoV (serology will be requested at the discretion of the investigator)
21. History of allergic reaction to Aminoglycoside antibiotics

6.3.3 Effective contraception for female volunteers

Female volunteers are required to use an effective form of contraception during the course of the study (i.e until their last follow up visit). As this is a Phase I, first-in-human, study there is no information about the effect of this vaccine on a foetus. Male subjects with female partners of child-bearing potential are not required to use barrier methods for the purposes of contraception whilst taking part in this study as the risk of excretion of the vaccine is negligible.

Acceptable forms of contraception for female volunteers include:

- Established use of oral, injected or implanted hormonal methods of contraception.
- Placement of an intrauterine device (IUD) or intrauterine system (IUS).
- Total abdominal hysterectomy

- Barrier methods of contraception (condom or occlusive cap with spermicide)
- Male sterilisation, if the vasectomised partner is the sole partner for the subject.
- True abstinence: when this is in line with the preferred and usual lifestyle of the subject. Periodic abstinence (e.g., calendar, ovulation, symptothermal, post-ovulation methods), declaration of abstinence for the duration of exposure to IMP, and withdrawal are not acceptable methods of contraception

6.3.4 Prevention of 'Over Volunteering'

Volunteers will be excluded from the study if they are concurrently involved in another trial. In order to check this, volunteers will be asked to provide their National Insurance or Passport number (if they are not entitled to a NI number) and will be registered on a national database of participants in clinical trials (www.tops.org.uk).

6.3.5 Criteria for postponement of vaccination

The following events constitute contraindications to administration of the vaccine at that point in time; if any one of these events occurs at the time scheduled for vaccination, the subject may be vaccinated at a later date, or withdrawn at the discretion of the Investigator.

- Acute disease at the time of vaccination. Acute disease is defined as the presence of a moderate or severe illness with or without fever. All vaccines can be administered to persons with a minor illness such as diarrhoea, mild upper respiratory infection with or without low-grade febrile illness, i.e. temperature of ≤37.5°C/99.5°F.
- Temperature of >37.5°C (99.5°F) at the time of vaccination.

6.3.6 Withdrawal of Volunteers

In accordance with the principles of the current revision of the Declaration of Helsinki and any other applicable regulations, a volunteer has the right to withdraw from the study at any time and for any reason, and is not obliged to give his or her reasons for doing so. The Investigator may withdraw the volunteer at any time in the interests of the volunteer's health and well-being. In addition the volunteer may withdraw/be withdrawn for any of the following reasons:

- Administrative decision by the Investigator.
- Ineligibility (either arising during the study or retrospectively, having been overlooked at screening).
- Significant protocol deviation.
- Volunteer non-compliance with study requirements.
- An AE, which requires discontinuation of the study involvement or results in inability to continue to comply with study procedures.

The reason for withdrawal will be recorded in the CRF. If withdrawal is due to an AE, appropriate follow-up visits or medical care will be arranged, with the agreement of the volunteer, until the AE has resolved, stabilised or a non-trial related causality has been assigned. Any volunteer who is

withdrawn from the study may be replaced, if that is possible within the specified time frame. The Chairman of LSC may recommend withdrawal of volunteers.

Any volunteer who fails to attend for two or more follow-up visits during the study will be deemed to have withdrawn from the study.

If a volunteer withdraws from the study, blood samples collected before their withdrawal from the trial will be used/ stored unless the volunteer specifically requests otherwise.

In all cases of subject withdrawal, excepting those of complete consent withdrawal, long-term safety data collection, including some procedures such as safety bloods, will continue as appropriate if subjects have received one or more vaccine doses.

6.4 Compliance with Dosing Regime

All doses in this vaccine study will be administered by the Investigator and recorded in the CRF. The study medication will be at no time in the possession of the volunteer and compliance will, therefore, not be an issue.

6.5 Pregnancy

Should a volunteer become pregnant during the trial, she will be followed up as other volunteers and in addition will be followed until pregnancy outcome. We will not routinely perform venepuncture in a pregnant volunteer.

7. CLINICAL PROCEDURES

This section describes the clinical procedures for evaluating study participants and follow-up after administration of study vaccine.

7.1 Study procedures

All volunteers will have the same schedule of clinic attendances and procedures as indicated in the schedules of attendance (Table 4). All subjects will receive the ChAdOx1 MERS vaccine, and undergo follow-up for a total of 52 weeks. The total volume of blood donated during the study will be 335mL. Additional visits or procedures may be performed at the discretion of the investigators, e.g., further medical history and physical examination, urine microscopy in the event of positive urinalysis or additional blood tests if clinically relevant.

7.2 Observations

Pulse, blood pressure and temperature will be measured at the time-points indicated in the schedule of procedures and may also be measured as part of a physical examination if indicated at other time-points.

7.3 Blood Tests and Urinalysis

Blood will be drawn for the following laboratory tests and processed:

1. At Oxford University Hospitals' NHS Trust using NHS standard procedures:

- **Haematology;** Full Blood Count
- **Biochemistry;** Sodium, Potassium, Urea, Creatinine, Albumin, Liver Function Tests (ALT, ALP, Bilirubin)
- **Diagnostic serology;** HBsAg, HCV antibodies, HIV antibodies (specific consent will be gained prior to testing blood for these blood-borne viruses)
- **Immunology;** Human Leukocyte Antigen (HLA) typing

Additional safety blood tests may be performed if clinically relevant at the discretion of the medically qualified investigators. These generally include, but are not limited to AST, GGT and a coagulation screen.

2. At University of Oxford research laboratories:

- **Exploratory Immunology;** Immunogenicity will be assessed by a variety of immunological assays. This may include antibodies to MERS-CoV Spike protein, ex vivo ELISpot assays for interferon gamma and flow cytometry assays, functional antibody assays and B cell analyses. Other exploratory immunological assays including cytokine analysis and other antibody assays, DNA analysis of genetic polymorphisms potentially relevant to vaccine immunogenicity and gene expression studies amongst others may be performed at the discretion of the Investigators.

3. **Urinalysis;** Urine will be tested for protein, blood and glucose at screening. For female volunteers only, urine will be tested for beta-human chorionic gonadotrophin (β-HCG) at screening and immediately prior to each vaccination.

Collaboration with other specialist laboratories in the UK, Europe and outside of Europe for further exploratory immunological tests may occur. This would involve the transfer of serum or plasma and/or PBMC to these laboratories, but these would remain anonymised. Informed consent for this will be gained from volunteers. Immunological assays will be conducted according to local SOPs.

Subjects will be informed that there may be leftover samples of their blood (after all testing for this study is completed), and that such samples may be stored indefinitely in the Oxford Vaccine Center Biobank for possible future research (exploratory immunology), including human DNA and RNA analyses to search for correlates of vaccine immunogenicity and efficacy. Subjects will be able to decide if they will permit such future use of any leftover samples. With the volunteers' informed consent, any leftover cells, urine and serum/plasma will be frozen indefinitely for future ethically approved research studies of MERS-CoV specific or vaccine-related responses. If a subject elects not to permit this, all of that subject's leftover samples will be discarded after the required period of storage to meet Good Clinical Practice (GCP) and regulatory requirements.

7.4 Study visits

The study visits and procedures will be undertaken by one of the clinical trials team. The procedures to be included in each visit are documented in the schedule of attendances (Table 4). Each visit is assigned a time-point and a window period, within which the visit will be conducted.

7.4.1 Screening visit

All potential volunteers will have a screening visit, which may take place up to 90 days prior to vaccination. Informed consent will be taken before screening, as described in section 6.2. If consent is obtained, the screening procedures indicated in the schedule of attendances will be undertaken. To avoid unnecessary additional venepuncture, if the appropriate blood test results for screening are available for the same volunteer from a screening visit for another Jenner Institute Clinical Trials group vaccine study, these results may be used for assessing eligibility (provided the results date is within the 3 months preceding enrolment in MERS001).

The subject's general practitioner will be contacted with the written permission of the subject after satisfactory screening as notification that the subject has volunteered for the study and to ascertain any significant medical history. During the screening the volunteers will be asked to provide their National Insurance or passport number so that this can be entered on to a national database which helps prevent volunteers from participating in more than one clinical trial simultaneously or over-volunteering for clinical trials (www.tops.org.uk).

Abnormal clinical findings from the urinalysis or blood tests at screening will be assessed by the lead clinician according to the relevant SOP. Abnormal blood tests following screening will be assessed according to site-specific laboratory adverse event grading tables which are filed in the trial master file (TMF) or the Investigator Site File (ISF). Any abnormal test result deemed clinically significant

may be repeated to ensure it is not a single occurrence. If an abnormal finding is deemed to be clinically significant, the volunteer will be informed and appropriate medical care arranged with the permission of the volunteer.

The eligibility of the volunteer will be reviewed at the end of the screening visit and again when all results from the screening visit have been considered. Decisions to exclude the volunteer from enrolling in the trial or to withdraw a volunteer from the trial will be at the discretion of the Investigator. If eligible, a day 0 visit will be scheduled for the volunteer to receive the vaccine.

7.4.2 Day 0: Enrolment and Vaccination Visit

Volunteers will not be considered enrolled in the study until they have received a vaccine. Before vaccination, the eligibility of the volunteer will be reviewed. Pulse, blood pressure and temperature will be observed and if necessary, a medical history and physical examination may be undertaken to determine need to postpone vaccination depending on criteria listed in section 6.3.5. Vaccinations will be administered as described below.

7.4.2.1 Vaccinations

Before each vaccination, the on-going eligibility of the volunteer will be reviewed. All vaccines will be administered intramuscularly according to SOP VC002 Vaccination as described below in section 8.4. The injection site will be covered with a sterile dressing and the volunteer will stay in the CCVTM for observation, in case of immediate adverse events. Observations will be taken 30 minutes after vaccination (+/- 5 minutes) and the sterile dressing removed and injection site inspected. Observations will also be taken at 60 minutes (+/- 10 minutes), before the volunteer leaves. An oral thermometer, tape measure and diary card (paper or electronic) will be given to each volunteer, with instructions on use, along with the emergency 24 hour telephone number to contact the on-call study physician if needed.

Diary cards will collect information on the timing and severity of the following solicited AEs:

Table 3. Solicited AEs as collected on post vaccination diary cards

Local solicited AEs	Systemic solicited AEs
Pain	Fever
Redness	Feverishness
Warmth	Joint pains
Itch	Muscle pains
	Fatigue
	Headache
	Nausea
	Malaise

Volunteers will be instructed on how to self-assess the severity of these AEs. There will also be space on the diary card to self-document unsolicited AEs, and whether medication was taken to relieve the symptoms.

7.4.2.2 Sequence of Enrolment and Vaccination of Volunteers

For safety reasons, the first volunteer in Group 1 will be vaccinated ahead of any other volunteers and the profile of adverse events will be reviewed after 48 hours (±24h) post vaccination. Provided there are no safety concerns, as assessed by the CI and the Chairman of LSC, another 2 volunteers will be vaccinated at the same dose after at least 48 hours has elapsed following the first volunteer being vaccinated and at least 1 hour apart from each other. An independent safety review will be conducted by the Chairman of LSC after vaccination of the first three volunteers. This review will include an assessment of the profile of adverse events and the results of safety blood tests at day 7 post vaccination. The CI and the Chairman of LSC will be asked to provide the decision on whether to proceed with vaccinations of the remaining participants in group 1 and the first volunteer to receive the next incremental dose in group 2. If there are no safety concerns, the remaining volunteers in Group 1 and the first volunteer in group 2 may be vaccinated.

Enrolment of the first volunteer in Group 2 will only proceed if the CI and Chairman of LSC assess the data from the first three vaccinees in Group 1 as indicating that it is safe to do so. The first subject in Group 2 will be vaccinated alone, and a 48 hour gap allowed before vaccinating further subjects in this group. Provided there are no safety concerns, as assessed by the CI and the Chairman of LSC, another 2 volunteers will be vaccinated at the same dose after at least 48 hours has elapsed following the first volunteer being vaccinated and at least 1 hour apart from each other. An independent safety review will be conducted by the Chairman of LSC after vaccination of the first three volunteers. This review will include an assessment of the profile of adverse events and the results of safety blood tests at day 7 post vaccination. The CI and the Chairman of LSC will be asked to provide the decision on whether to proceed with vaccinations of the remaining participants in group 2 and the first volunteer to receive the next incremental dose in group 3. If there are no safety concerns, the remaining volunteers in Group 2 and the first volunteer in group 3 may be vaccinated.

Enrolment of the first volunteer in Group 3 will only proceed if the CI and Chairman of LSC assess the data from the first three vaccinees in Group 2 as indicating that it is safe to do so. The first subject in Group 3 will be vaccinated alone, and a 48 hour gap allowed before vaccinating further subjects in this group. Provided there are no safety concerns, as assessed by the CI and the Chairman of LSC, another 2 volunteers will be vaccinated at the same dose after at least 48 hours has elapsed following the first volunteer being vaccinated and at least 1 hour apart from each other. An independent safety review will be conducted by the Chairman of LSC after vaccination of the first three volunteers. This review will include an assessment of the profile of adverse events and the results of safety blood tests at day 7 post vaccination. The CI and the Chairman of LSC will be asked to provide the decision on whether to proceed with vaccinations of the remaining participants in group 3. If there are no safety concerns, the remaining volunteers in Group 3 may be vaccinated.

7.4.3 Subsequent visits: days 2, 7, 14, 28, 56, 182 and 364.

Follow-up visits will take place 48 hours (±24h), 7 days (± 2 days), 14 days (± 3 days), 28 days (± 3 days), 56 days (± 7 days), 182 (± 14 days) and 364 (± 30 days) after vaccination. Volunteers will be assessed for local and systemic adverse events, interim history, physical examination, review of diary cards (paper or electronic) and blood tests at these time points as detailed in the schedule of attendances. Blood will also be taken for exploratory immunology purposes.

If volunteers experience adverse events (laboratory or clinical), which the investigator (physician), CI and/or Chairman of LSC determine necessary for further close observation, the volunteer may be admitted to an NHS hospital for observation and further medical management under the care of the Consultant on call.

Table 4. Schedule of attendances

Attendance Number	1[S]	2	3	4	5	6	7	8	9
Timeline** (days)	≤ 90	0	2	7	14	28	56	182	364
Time window (days)			±1	±2	±3	±3	±7	±14	±30
Informed Consent	X								
Review contraindications, inclusion and exclusion criteria	X	X							
Vaccination		X							
Vital signs^	X	X	X	X	X	X	X	X	X
Ascertainment of adverse events		X	X	X	X	X	X	X	X
Diary cards provided		X							
Diary cards collected						X			
Medical History, Physical Examination	X	(X)	(X)	(X)	(X)	(X)	(X)	(X)	(X)
Biochemistry[$], Haematology (mL)	5	5	5	5		5			
Exploratory immunology[£] (mL)		50			50	50	50	50	50
Urinalysis	X								
Urinary β–HCG (women only)	X	X							
HLA typing (mL)		4							
HBsAg, HCV Ab, HIV serology (mL)	5								
Blood volume per visit	10	59		5	50	55	50	50	50
Cumulative blood volume[%]	10	69	74	79	129	184	234	284	334

S = screening visit; (X) = if considered necessary ^ = Vital signs includes pulse, blood pressure and temperature; $ = Biochemistry will include Sodium, Potassium, Urea, Creatinine, Albumin and Liver function tests. £ = Exploratory immunology includes antibodies to MERS-CoV S, ex vivo interferon-gamma ELISpot responses to MERS-CoV S
** Timeline is approximate only. Exact timings of visits relate to the day on enrolment, ie, each visit must occur at indicated number of days after enrolment ± time window.

% Cumulative blood volume for Oxford volunteers if blood taken as per schedule, and excluding any repeat safety blood test that may be necessary.

8. INVESTIGATIONAL PRODUCTS

The following vaccinations will be given in this study:

1. ChAdOx1 MERS 5×10^9vp
2. ChAdOx1 MERS 2.5×10^{10}vp
3. ChAdOx1 MERS 5×10^{10}vp

8.1. Manufacturing and Presentation

8.1.1 Description of ChAdOx1 MERS

ChAdOx1 MERS vaccine consists of the replication-deficient simian adenovirus vector ChAdOx1, containing the structural surface glycoprotein (Spike protein) antigens of the MERS-CoV expressed from the strong CMV IE promoter.

8.1.2. ChAdOx1 MERS formulation and packaging

ChAdOx1 MERS is manufactured in formulation buffer at a concentration of 1.74×10^{11} vp/mL . The drug product is filled into 2mL glass vials with a 13 mm grey bromobutyl rubber freeze-dry stopper (CE Marked, supplied by Adelphi Tubes) and a 13 mm aluminium seal. The nitrogen filled vials are supplied sterile. The containers and closures are tested for compliance with defined specifications. The vials are made from Ph Eur Type 1 glass.

8.2 Supply

ChAdOx1 MERS has been formulated and vialed under Good Manufacturing Practice conditions at the Clinical Biomanufacturing Facility (CBF), University of Oxford. At the CBF the vaccine will be certified and labelled for the trial by a Qualified Person (QP) before transfer to the clinical site.

8.3 Storage

The vaccine is stored at nominal -80°C in a locked freezer, at the clinical site. All movements of the study vaccines will be documented in accordance with existing standard operating procedure (SOP). Vaccine accountability, storage, shipment and handling will be in accordance with relevant SOPs and forms.

8.4 Administration of Investigational Medicinal Products

On vaccination day, ChAdOx1 MERS will be allowed to thaw to room temperature and will be administered within 1 hour of removal from the freezer. The vaccine will be administered intramuscularly into the deltoid of the non-dominant arm (preferably). All volunteers will be observed in the unit for 1 hour (±10 minutes) after vaccination. During administration of the investigational products, Advanced Life Support drugs and resuscitation equipment will be immediately available for the management of anaphylaxis. Vaccination will be performed and the IMPs handled according to the relevant SOPs.

8.5 Minimising environmental contamination with genetically modified organisms (GMO)

The study will be performed in accordance with UK Genetically Modified Organisms (Contained Use) Regulations (2014). In order to minimise dissemination of the recombinant vectored vaccine virus into the environment, inoculation sites will be covered with a dressing after immunisation. This should absorb any virus that may leak out through the needle track. The dressing will be removed from the injection site after 30 minutes (+15/- 5 minutes) and will be disposed as GMO waste by autoclaving.

9. ASSESSMENT OF SAFETY

Safety will be assessed by the frequency, incidence and nature of adverse events and serious adverse events arising during the study.

9.1 Definitions

9.1.1 Adverse Event (AE)

An AE is any untoward medical occurrence in a volunteer, which may occur during or after administration of an Investigational Medicinal Product (IMP) and does not necessarily have a causal relationship with the intervention. An AE can therefore be any unfavourable and unintended sign (including an abnormal laboratory finding), symptom or disease temporally associated with the study intervention, whether or not considered related to the study intervention.

9.1.2 Adverse Reaction (AR)

An AR is any untoward or unintended response to an IMP. This means that a causal relationship between the IMP and an AE is at least a reasonable possibility, i.e., the relationship cannot be ruled out. All cases judged by the reporting medical Investigator as having a reasonable suspected causal relationship to an IMP (i.e. possibly, probably or definitely related to an IMP) will qualify as adverse reactions.

9.1.3 Unexpected Adverse Reaction

An adverse reaction, the nature or severity of which is not consistent with the applicable product information (e.g., IB for an unapproved IMP).

9.1.4 Serious Adverse Event (SAE)

An SAE is an AE that results in any of the following outcomes, whether or not considered related to the study intervention.

- Death
- Life-threatening event (i.e., the volunteer was, in the view of the Investigator, at immediate risk of death from the event that occurred). This does not include an AE that, if it occurred in a more severe form, might have caused death.
- Persistent or significant disability or incapacity (i.e., substantial disruption of one’s ability to carry out normal life functions).
- Hospitalisation, regardless of length of stay, even if it is a precautionary measure for continued observation. Hospitalisation (including inpatient or outpatient hospitalisation for an elective procedure) for a pre-existing condition that has not worsened unexpectedly does not constitute a serious AE.
- An important medical event (that may not cause death, be life threatening, or require hospitalisation) that may, based upon appropriate medical judgment, jeopardise the volunteer and/or require medical or surgical intervention to prevent one of the outcomes listed above. Examples of such medical events include allergic reaction requiring intensive treatment in an emergency room or clinic, blood dyscrasias, or convulsions that do not result in inpatient hospitalisation.

- Congenital anomaly or birth defect.

9.1.5 Serious Adverse Reaction (SAR)

An adverse event (expected or unexpected) that is both serious and, in the opinion of the reporting Investigator or Sponsors, believed to be possibly, probably or definitely due to an IMP or any other study treatments, based on the information provided.

9.1.6 Suspected Unexpected Serious Adverse Reaction (SUSAR)

A serious adverse reaction, the nature and severity of which is not consistent with the information about the medicinal product in question set out in the IB or Summary of Product Characteristics (SmPC).

9.2 Foreseeable Adverse Reactions:

The foreseeable ARs following vaccination with ChAdOx1 MERS include injection site pain, erythema, warmth, swelling, pruritus, myalgia, arthralgia, headache, fatigue, fever, feverishness, malaise and nausea.

9.3 Expected Serious Adverse Events

No serious adverse events are expected in this study.

9.4 Causality Assessment

For every AE, an assessment of the relationship of the event to the administration of the vaccine will be undertaken by the CI-delegated clinician. An intervention-related AE refers to an AE for which there is a probable or definite relationship to administration of a vaccine. An interpretation of the causal relationship of the intervention to the AE in question will be made, based on the type of event; the relationship of the event to the time of vaccine administration; and the known biology of the vaccine therapy (Table 5). Alternative causes of the AE, such as the natural history of pre-existing medical conditions, concomitant therapy, other risk factors and the temporal relationship of the event to vaccination will be considered and investigated. Causality assessment will take place during planned safety reviews, interim analyses (e.g. if a holding or stopping rule is activated) and at the final safety analysis, except for SAEs, which should be assigned by the reporting investigator.

Table 5. Guidelines for assessing the relationship of vaccine administration to an AE.

0	No Relationship	No temporal relationship to study product ***and***<br>Alternate aetiology (clinical state, environmental or other interventions); ***and***<br>Does not follow known pattern of response to study product
1	Unlikely	Unlikely temporal relationship to study product ***and***<br>Alternate aetiology likely (clinical state, environmental or other interventions) ***and***<br>Does not follow known typical or plausible pattern of response to study product.
2	Possible	Reasonable temporal relationship to study product; ***or***<br>Event not readily produced by clinical state, environmental or other interventions; ***or***<br>Similar pattern of response to that seen with other vaccines
3	Probable	Reasonable temporal relationship to study product; ***and***<br>Event not readily produced by clinical state, environment, or other interventions ***or***<br>Known pattern of response seen with other vaccines
4	Definite	Reasonable temporal relationship to study product; ***and***<br>Event not readily produced by clinical state, environment, or other interventions; ***and***<br>Known pattern of response seen with other vaccines

9.5 Reporting Procedures for All Adverse Events (see SOP VC027)

All local and systemic AEs occurring in the 28 days following each vaccination observed by the Investigator or reported by the volunteer, whether or not attributed to study medication, will be recorded (excluding those expected consequences from venepuncture, described in section 5.3). Recording and reporting of all AEs will take place as detailed in SOP VC027. All AEs that result in a volunteer's withdrawal from the study will be followed up until a satisfactory resolution occurs, or until a non-study related causality is assigned (if the volunteer consents to this). Serious adverse events (SAEs) will be collected throughout the entire trial period.

9.5.1 Reporting Procedures for Serious AEs (see SOP OVC005 Safety Reporting)

In order to comply with current regulations on serious adverse event reporting to regulatory authorities, the event will be documented accurately and notification deadlines respected. SAEs will be reported on the SAE forms to members of the study team immediately the Investigators become aware of their occurrence, as described in SOP OVC005. Copies of all reports will be forwarded for review to the Chief Investigator (as the Sponsor's representative) within 24 hours of the Investigator being aware of the suspected SAE. The Chairman of LSC will be notified of SAEs that are deemed possibly, probably or definitely related to study interventions; the Chairman of LSC will be notified immediately (within 24 hours) of the Investigators' being aware of their occurrence. SAEs will not normally be reported immediately to the ethical committee(s) unless there is a clinically important increase in occurrence rate, an unexpected outcome, or a new event that is likely to affect safety of trial volunteers, at the discretion of the Chief Investigator and/or Chairman of LSC. In addition to the

expedited reporting above, the Investigator shall include all SAEs in the annual Development Safety Update Report (DSUR) report.

9.5.2 Reporting Procedures for SUSARS

The Chief Investigator will report all SUSARs to the MHRA and ethical committee(s) within required timelines (15 days for all SUSARs, unless life threatening in which case 7 days, with a final report within a further 8 days (total 15). The Chief Investigator will also inform all Investigators concerned of relevant information about SUSARs that could adversely affect the safety of participants. All SUSARs and deaths occurring during the study will be reported to the Sponsor. For all deaths, available autopsy reports and relevant medical reports will be made available for reporting to the relevant authorities.

9.5.3 Development Safety Update Report

A Development Safety Update Report (DSUR) will be submitted by the Sponsor to the competent authority and ethical committee on the anniversary of the first approval date from the regulatory authority for each IMP.

9.6 Assessment of severity

The severity of clinical and laboratory adverse events will be assessed according to the scales in Tables 6-8, also described in the SOP VC027.

Table 6. Severity grading criteria for local adverse events.

Adverse Event	Grade	Intensity
Erythema at injection site*	1	>3 - ≤50 mm
	2	>50 - ≤100 mm
	3	>100 mm
Swelling at injection site	1	>3 - ≤20 mm
	2	>20 - ≤50 mm
	3	>50 mm
Ulceration/necrosis of skin at injection site	1	-
	2	-
	3	Any

*erythema or swelling ≤3mm is an expected consequence of skin puncture and will therefore not be considered an adverse event.

Table 7. Severity grading criteria for physical observations

	Grade 1 (mild)	Grade 2 (moderate)	Grade 3 (severe)
Fever (oral)	37.6°C - 38.0°C	38.1°C – 39.0°C	>39.0°C
Tachycardia (bpm)*	101 - 115	116 – 130	>130
Bradycardia (bpm)**	50 – 54	40 – 49	<40
Systolic hypertension (mmHg)	141 - 159	160 – 179	≥180
Systolic hypotension (mmHg)***	85 - 89	80 – 84	<80
Diastolic hypertension (mmHg)	91 - 99	100 – 109	≥110

*Taken after ≥10 minutes at rest

**Use clinical judgement when characterising bradycardia among some healthy subject populations, for example, conditioned athletes.

***Only if symptomatic (e.g. dizzy/ light-headed)

Table 8. Severity grading criteria for local and systemic AEs.

GRADE 0	None: Symptom not experienced
GRADE 1	Mild: Short-lived or mild symptoms; medication may be required. No limitation to usual activity
GRADE 2	Moderate: Mild to moderate limitation in usual activity. Medication may be required.
GRADE 3	Severe: Considerable limitation in activity. Medication or medical attention required.

9.7 Procedures to be followed in the event of abnormal findings

Laboratory parameters for inclusion/exclusion in the trial will be considered on an individual basis, with investigator discretion for interpretation of results and the need for repeated tests. Laboratory adverse events will be assessed using the tables as detailed in SOP VC027. Abnormal clinical findings from medical history, examination or blood tests will be assessed as to their clinical significance throughout the trial. If a test is deemed clinically significant, it may be repeated, to ensure it is not a single occurrence. If a test remains clinically significant, the volunteer will be informed and appropriate medical care arranged as appropriate and with the permission of the volunteer. Decisions to exclude the volunteer from enrolling in the trial or to withdraw a volunteer from the trial will be at the discretion of the Investigator.

9.8 Local Safety Committee

A Local Safety Committee (LSC) will be appointed to provide real-time safety oversight. The LSC will review SAEs deemed possibly, probably or definitely related to study interventions. The LSC will be notified within 24 hours of the Investigators' being aware of their occurrence. The LSC has the power to place the study on hold if deemed necessary following a study intervention-related SAE. At the

time of writing the LSC will be chaired by Dr Brian Angus, a Clinical Tutor in Medicine, Honorary Consultant Physician and Director, Centre for Tropical Medicine at the University of Oxford, . There will be a minimum of two other appropriately qualified committee members.

The chair of the LSC may be contacted for advice and independent review by the Investigator or trial Sponsor in the following situations:

- Following any SAE deemed to be possibly, probably, or definitely related to a study intervention.
- Any other situation where the Investigator or trial Sponsor feels independent advice or review is important.

9.8.1 Interim Safety Reviews

Interim safety reviews with the Chairman of the LSC are scheduled during the enrolment of the first volunteers in each group and prior to dose escalations, as outlined in section 7.4.2.2.

The safety profile of the IMP will be assessed on an on-going basis by the Investigators with communication to the LSC as necessary. The Chief Investigator and relevant Investigators (as per the trial delegation log) will also review safety issues and SAEs as they arise.

9.9 Safety Stopping/Holding Rules

Safety holding rules have been developed considering the fact that this is a first-in-human dose escalation study.

'Solicited adverse events' are those listed as foreseeable adverse events in section 9.3 of the protocol, occurring within the first 7 days after vaccination (day of vaccination and six subsequent days). 'Unsolicited adverse events' are adverse events other than the foreseeable AEs occurring within the first 7 days, or any AEs occurring after the first 7 days after vaccination.

9.9.1 Group holding rules

For safety reasons the first volunteer to receive a new vaccine dose in Groups 1-3 will be vaccinated alone and we will wait 48 hours before vaccinating subsequent volunteers. Two further volunteers may be vaccinated 48 hours after the first, and then at least another 48 hours gap will be left before vaccinating the rest of the volunteers receiving the same dose of the vaccine.

- **Solicited local adverse events:**
    - If 2 or more vaccinations in a group are followed by the same Grade 3 solicited local adverse event beginning within 2 days after vaccination (day of vaccination and one subsequent day) and persisting at Grade 3 for >48 hrs.

- **Solicited systemic adverse events:**
    - If 2 or more vaccinations in a group are followed by the same Grade 3 solicited systemic adverse event beginning within 2 days after vaccination (day of vaccination and one subsequent day) and persisting at Grade 3 for >48 hrs.

- **Unsolicited adverse events:**
    - If 2 or more vaccinations in a group are followed by the same Grade 3 unsolicited adverse event (including the same laboratory adverse event) that is considered possibly, probably or definitely related to vaccination and persists at Grade 3 for > 48hrs.

- **A serious adverse event considered possibly, probably or definitely related to vaccination occurs**

- **Death occurs**

- **A life-threatening reaction occurs**

If a holding rule has been met we will inform the regulatory authority; following an internal safety review, if it is deemed appropriate to restart dosing, a request to restart dosing with pertinent data must be submitted to the regulatory authority as a request for a substantial amendment. The internal safety review will consider:

- The relationship of the AE or SAE to the vaccine.
- The relationship of the AE or SAE to the vaccine dose, or other possible causes of the event.
- If appropriate, additional screening or laboratory testing for other volunteers to identify those who may develop similar symptoms and alterations to the current Participant Information Sheet (PIS) are discussed.
- New, relevant safety information from ongoing research programs on the various components of the vaccine.

The sponsor, local ethics committee and vaccine manufacturers will also be notified if a holding rule is activated or released.

As per section 6.3.5, if a volunteer has an acute illness (moderate or severe illness with or without fever) or a fever (oral temperature greater than 37.5°C) at the scheduled time of administration of investigational product, the volunteer will not receive the vaccine at that time. The vaccine may be administered to that volunteer at a later date within the time window specified in the protocol (see Table 4) or they may be withdrawn from the study at the discretion of the Investigator.

All vaccinated volunteers will be followed for safety until the end of their planned participation in the study or until resolution or stabilisation (if determined to be chronic sequelae) of their AEs, providing they consent to this.

In addition to these pre-defined criteria, the study can be put on hold upon advice of the Local Safety Monitor, Chief Investigator, Study Sponsor, Regulatory Authority, Ethical Committee(s) or Local Safety Committee, for any single event or combination of multiple events which, in their professional opinion, jeopardise the safety of the volunteers or the reliability of the data.

10. STATISTICS

This is a descriptive safety study, where volunteers will be vaccinated with a single dose of ChAdOx1 MERS. Twenty-four volunteers will be vaccinated in total. This sample size should allow an estimation to be made of the frequency and magnitude of outcome measures, rather than aiming to obtain statistical significance for differences between groups. Safety data will be presented according to frequency, severity and duration of adverse events.

The primary analysis for immunogenicity will be to assess the difference in magnitude of MERS-CoV specific T-cell and antibody responses between the groups. We will assess vaccine immunogenicity by comparing the change in these immunological parameters from baseline to different time points.

Statistical analysis will be conducted according to local SOPs and an agreed Statistical Analysis Plan when required.

Sample Size Selection

This is a descriptive phase I first in human trial that will balance the safety of volunteers with the aims to assess the vaccine's safety profile and immunogenicity after selected doses of the vaccines. The primary dose comparison will be between Groups 1, 2 and 3, which will have 6-9 subjects each. MERS-CoV S-specific immunogenicity will be the key immunological readout assessed by a variety of immunological assays.

11. DATA MANAGEMENT

11.1 Data Handling

The Chief Investigator will be responsible for all data that accrues from the study. The data will be entered into the volunteers' CRFs in a paper and/or electronic format (using OpenClinica™ database). Electronic data will be stored on secure servers which are outsourced by OpenClinica™. Data will be entered in a web browser on PCs in the CCVTM building and then transferred to the OpenClinica Database by encrypted (Https) transfer. OpenClinica™ meets FDA part 11B standards. This includes safety data, laboratory data (both clinical and immunological) and outcome data.

Adverse event data will also be entered onto electronic or paper diaries by the volunteer

11.2 Record Keeping

The Investigators will maintain appropriate medical and research records for this trial, in compliance with GCP and regulatory and institutional requirements for the protection of confidentiality of volunteers. The Chief Investigator, co-Investigators and clinical research nurses will have access to records. The Investigators will permit authorised representatives of the Sponsor(s), as well as ethical and regulatory agencies to examine (and when required by applicable law, to copy) clinical records for the purposes of quality assurance reviews, audits and evaluation of the study safety and progress.

11.3 Source Data and Case Report Forms (CRFs)

All protocol-required information will be collected in CRFs designed by the Investigator. All source documents will be filed in the CRF. Source documents are original documents, data, and records from which the volunteer's CRF data are obtained. For this study, these will include, but are not limited to, volunteer consent form, blood results, GP response letters, laboratory records, diaries, and correspondence. In the majority of cases, CRF entries will be considered source data as the CRF is the site of the original recording (i.e. there is no other written or electronic record of data). In this study this will include, but is not limited to medical history, medication records, vital signs, physical examination records, urine assessments, blood results, adverse event data and details of vaccinations. All source data and volunteer CRFs will be stored securely.

11.4 Data Protection

The study protocol, documentation, data and all other information generated will be held in strict confidence. No information concerning the study or the data will be released to any unauthorised third party, without prior written approval of the sponsor.

11.5 Data Quality

Data collection tools will undergo appropriate validation to ensure that data is collected accurately and completely. Datasets provided for analysis will be subject to quality control processes to ensure analysed data is a true reflection of the source data.

Trial data will be managed in compliance with local data management SOPs (including the overarching SOP OVC007 Data and Database Management). If additional, study specific information is required, an approved Data Management Plan will be implemented.

The trial will comply with the General Data Protection Regulation (GDPR) and Data Protection Act 2018, which requires data to be anonymised as soon as it is practical to do so.

12. QUALITY CONTROL AND QUALITY ASSURANCE PROCEDURES

12.1 Investigator procedures

Approved site-specific standard operating procedures (SOPs) will be used at all clinical and laboratory sites.

12.2 Monitoring

Monitoring will be performed according to ICH GCP by Clinical Trials Research Governance (CTRG). Following written SOPs, the monitors will verify that the clinical trial is conducted and data are generated, documented and reported in compliance with the protocol, GCP and the applicable regulatory requirements. The Investigator sites will provide direct access to all trial related source data/documents and reports for the purpose of monitoring and auditing by the Sponsor and inspection by local and regulatory authorities.

12.3 Protocol deviation

Any deviations from the protocol will be documented in a protocol deviation form and filed in the trial master file. Each deviation will be assessed as to its impact on volunteer safety and study conduct. Significant deviations will be listed in the end of study report.

12.4 Audit & inspection

The QA manager conducts systems based internal audits to check that trials are being conducted according to local procedures and in compliance with GCP and applicable regulations.

The Sponsor, trial sites, and ethical committee(s) may carry out audit to ensure compliance with the protocol, GCP and appropriate regulations.

GCP inspections may also be undertaken by the MHRA to ensure compliance with protocol and the Medicines for Human Use (Clinical Trials) Regulations 2004, as amended. The Sponsor will assist in any inspections and will support the response to the MHRA as part of the inspection procedure.

13. SERIOUS BREACHES

The Medicines for Human Use (Clinical Trials) Regulations contain a requirement for the notification of "serious breaches" to the MHRA within 7 days of the Sponsor becoming aware of the breach.

A serious breach is defined as “A breach of GCP or the trial protocol which is likely to effect to a significant degree

(a) the safety or physical or mental integrity of the subjects of the trial; or

(b) the scientific value of the trial”.

In the event that a serious breach is suspected the Sponsor will be informed within one working day.

14. ETHICS AND REGULATORY CONSIDERATIONS

14.1 Declaration of Helsinki

The Investigators will ensure that this study is conducted according to the principles of the current revision of the Declaration of Helsinki.

14.2 Guidelines for Good Clinical Practice

The Investigators will ensure that this study is conducted in full conformity with the Good Clinical Practice (GCP).

14.3 Approvals

The protocol, informed consent form, participant information sheet and any proposed advertising material will be submitted to an appropriate Research Ethics Committee (REC), HRA (where required), regulatory authorities (MHRA in the UK), and host institution(s) for written approval.

The Investigator will submit and, where necessary, obtain approval from the above parties for all substantial amendments to the original approved documents.

No substantial amendments to this protocol will be made without consultation with, and agreement of, the Sponsor. Any substantial amendments to the trial that appear necessary during the course of the trial must be discussed by the Investigator and Sponsor concurrently. If agreement is reached concerning the need for an amendment, it will be produced in writing by the Chief Investigator and will be made a formal part of the protocol following ethical and regulatory approval.

The Investigator is responsible for ensuring that changes to an approved trial, during the period for which regulatory and ethical committee(s) approval has already been given, are not initiated without regulatory and ethical committee(s)' review and approval except to eliminate apparent immediate hazards to the subject.

14.4 Volunteer Confidentiality

All data will be anonymised: volunteer data will be identified by a unique study number in the CRF and database. A separate confidential file containing identifiable information will be stored in a secured location in accordance with the Data Protection Act 1998. Only the Sponsor representative, Investigators, the clinical monitor, the REC and the MHRA will have access to the records. Photographs taken of vaccination sites (if required, with the volunteer's written, informed consent) will not include the volunteer's face and will be identified by the date, trial code and subject's unique identifier. Once developed, photographs will be stored as confidential records, as above. This material may be shown to other professional staff, used for educational purposes, or included in a scientific publication.

15. FINANCING AND INSURANCE

15.1 Financing

The study is funded by the UK Department of Health.

15.2 Insurance

The University has a specialist insurance policy in place which would operate in the event of any participant suffering harm as a result of their involvement in the research (Newline Underwriting Management Ltd, at Lloyd's of London).

15.3 Compensation

Volunteers will be compensated for their time and for the inconvenience caused by procedures. They will be compensated £25 for attending the screening visit. For all other trial visits as outlined in Table 4, compensation will be calculated according to the following:

- Travel expenses:
    - £10 per visit. Where travel expenses are greater than £10 per visit because the volunteer lives outside the city of the trial site, the volunteer will be given further reimbursement to meet the cost of travel necessary for study visits.

- Inconvenience of blood tests:
    - £10 per blood donation

- Time required for visit:
    - £20 per hour

The total amount compensated will be approximately £375 depending on the exact number of visits and whether any repeat or additional visits are necessary.

16. PUBLICATION POLICY

The Investigators will be involved in reviewing drafts of the manuscripts, abstracts, press releases and any other publications arising from the study. Data from the study may also be used as part of a thesis for a PhD or MD.

17. REFERENCES

1. WHO. WHO MERS-CoV Global Summary and risk assessment. WHO; 2016.

2. Badawi A, Ryoo SG. Prevalence of comorbidities in the Middle East respiratory syndrome coronavirus (MERS-CoV): a systematic review and meta-analysis. International Journal of Infectious Diseases. 2016;49:129-33.

3. Assiri A, Al-Tawfiq JA, Al-Rabeeah AA, Al-Rabiah FA, Al-Hajjar S, Al-Barrak A, et al. Epidemiological, demographic, and clinical characteristics of 47 cases of Middle East respiratory syndrome coronavirus disease from Saudi Arabia: a descriptive study. Lancet Infect Dis. 2013;13(9):752-61.

4. WHO. Fact Sheet: Middle East respiratory syndrome coronavirus (MERS-CoV). WHO; 2015.

5. Mackay IM, Arden KE. MERS coronavirus: diagnostics, epidemiology and transmission. Virology journal. 2015;12:222.

6. Cohen J. Unfilled Vials. Science. 2016;351(6268):16-9.

7. Antrobus RD, Coughlan L, Berthoud TK, Dicks MD, Hill AV, Lambe T, et al. Clinical assessment of a novel recombinant simian adenovirus ChAdOx1 as a vectored vaccine expressing conserved Influenza A antigens. Mol Ther. 2014;22(3):668-74.

8. Modjarrad K. MERS-CoV vaccine candidates in development: The current landscape. Vaccine. 2016;34(26):2982-7.

9. Alharbi NK. Vaccines against Middle East respiratory syndrome coronavirus for humans and camels. Reviews in medical virology. 2016.

10. Haagmans BL, van den Brand JM, Raj VS, Volz A, Wohlsein P, Smits SL, et al. An orthopoxvirus-based vaccine reduces virus excretion after MERS-CoV infection in dromedary camels. Science. 2016;351(6268):77-81.

11. Wang Q, Wong G, Lu G, Yan J, Gao GF. MERS-CoV spike protein: Targets for vaccines and therapeutics. Antiviral research. 2016;133:165-77.

12. Du L, Tai W, Zhou Y, Jiang S. Vaccines for the prevention against the threat of MERS-CoV. Expert review of vaccines. 2016;15(9):1123-34.

13. Bett AJ, Haddara W, Prevec L, Graham FL. An efficient and flexible system for construction of adenovirus vectors with insertions or deletions in early regions 1 and 3. Proceedings of the National Academy of Sciences of the United States of America. 1994;91(19):8802-6.

14. Top FH, Jr., Grossman RA, Bartelloni PJ, Segal HE, Dudding BA, Russell PK, et al. Immunization with live types 7 and 4 adenovirus vaccines. I. Safety, infectivity, antigenicity, and potency of adenovirus type 7 vaccine in humans. The Journal of infectious diseases. 1971;124(2):148-54.

15. Grabenstein JD, Pittman PR, Greenwood JT, Engler RJ. Immunization to protect the US Armed Forces: heritage, current practice, and prospects. Epidemiol Rev. 2006;28:3-26.

16. Farina SF, Gao GP, Xiang ZQ, Rux JJ, Burnett RM, Alvira MR, et al. Replication-defective vector based on a chimpanzee adenovirus. J Virol. 2001;75(23):11603-13.

17. Bruna-Romero O, Gonzalez-Aseguinolaza G, Hafalla JC, Tsuji M, Nussenzweig RS. Complete, long-lasting protection against malaria of mice primed and boosted with two distinct viral vectors expressing the same plasmodial antigen. Proceedings of the National Academy of Sciences of the United States of America. 2001;98(20):11491-6.

18. Tatsis N, Tesema L, Robinson ER, Giles-Davis W, McCoy K, Gao GP, et al. Chimpanzee-origin adenovirus vectors as vaccine carriers. Gene Ther. 2006;13(5):421-9.

19. Dicks MD, Spencer AJ, Edwards NJ, Wadell G, Bojang K, Gilbert SC, et al. A novel chimpanzee adenovirus vector with low human seroprevalence: improved systems for vector derivation and comparative immunogenicity. PloS one. 2012;7(7):e40385.

20. Alharbi NK, Padron-Regalado E, Thompson CP, Kupke A, Wells D, Sloan MA, et al. ChAdOx1 and MVA based vaccine candidates against MERS-CoV elicit neutralising antibodies and cellular immune responses in mice. Vaccine. 2017;35(30):3780-8.

21. Antrobus RD, Berthoud TK, Mullarkey CE, Hoschler K, Coughlan L, Zambon M, et al. Coadministration of seasonal influenza vaccine and MVA-NP+M1 simultaneously achieves potent humoral and cell-mediated responses. Mol Ther. 2014;22(1):233-8.

22. Antrobus RD, Lillie PJ, Berthoud TK, Spencer AJ, McLaren JE, Ladell K, et al. A T cell-inducing influenza vaccine for the elderly: safety and immunogenicity of MVA-NP+M1 in adults aged over 50 years. PloS one. 2012;7(10):e48322.

23. Berthoud TK, Hamill M, Lillie PJ, Hwenda L, Collins KA, Ewer KJ, et al. Potent CD8+ T-cell immunogenicity in humans of a novel heterosubtypic influenza A vaccine, MVA-NP+M1. Clin Infect Dis. 2011;52(1):1-7.

24. Ewer KJ, O'Hara GA, Duncan CJ, Collins KA, Sheehy SH, Reyes-Sandoval A, et al. Protective CD8+ T-cell immunity to human malaria induced by chimpanzee adenovirus-MVA immunisation. Nature communications. 2013;4:2836.

25. Borthwick N, Ahmed T, Ondondo B, Hayes P, Rose A, Ebrahimsa U, et al. Vaccine-elicited human T cells recognizing conserved protein regions inhibit HIV-1. Molecular therapy : the journal of the American Society of Gene Therapy. 2014;22(2):464-75.

26. Swadling L, Capone S, Antrobus RD, Brown A, Richardson R, Newell EW, et al. A human vaccine strategy based on chimpanzee adenoviral and MVA vectors that primes, boosts, and sustains functional HCV-specific T cell memory. Sci Transl Med. 2014;6(261):261ra153.

27. Green CA, Scarselli E, Sande CJ, Thompson AJ, de Lara CM, Taylor KS, et al. Chimpanzee adenovirus- and MVA-vectored respiratory syncytial virus vaccine is safe and immunogenic in adults. Science translational medicine. 2015;7(300):300ra126.

28. Ewer K, Rampling T, Venkatraman N, Bowyer G, Wright D, Lambe T, et al. A Monovalent Chimpanzee Adenovirus Ebola Vaccine Boosted with MVA. The New England journal of medicine. 2016;374(17):1635-46.

29. Jones KE, Patel NG, Levy MA, Storeygard A, Balk D, Gittleman JL, et al. Global trends in emerging infectious diseases. Nature. 2008;451(7181):990-3.

30. Warimwe GM, Gesharisha J, Carr BV, Otieno S, Otingah K, Wright D, et al. Chimpanzee Adenovirus Vaccine Provides Multispecies Protection against Rift Valley Fever. Scientific reports. 2016;6:20617.
